# Age‐related changes in murine myometrial transcript profile are mediated by exposure to the female sex hormones

**DOI:** 10.1111/acel.12406

**Published:** 2015-10-21

**Authors:** Hsu P. Chong, Yolande Cordeaux, Yorain Sri Ranjan, Sylvia Richardson, Benoit Liquet, Gordon C. S. Smith, David Stephen Charnock‐Jones

**Affiliations:** ^1^ Department of Obstetrics and Gynaecology & NIHR Biomedical Research CentreUniversity of CambridgeThe Rosie HospitalRobinson WayCambridgeCB2 0SWUK; ^2^ MRC Biostatistics UnitCambridge Institute of Public HealthCambridgeCB2 0SRUK; ^3^ Division of Reproductive HealthWarwick Medical SchoolUniversity of WarwickWarwickUK; ^4^ School of Mathematics and PhysicsThe University of QueenslandBrisbaneQldAustralia

**Keywords:** steroid, myometrium, aging, labor, IRF7

## Abstract

In humans, the risk of operative first delivery increases linearly with maternal age. We previously hypothesized that prolonged, cyclical, prepregnancy exposure to estrogen and progesterone contributes to uterine aging. Here, we test this hypothesis. Myometrium was obtained from four groups of virgin mice: (i) 10‐ to 12‐week‐ and 28‐ to 30‐week‐old mice; (ii) 10‐ to 12‐week‐ and 38‐ to 40‐week‐old mice; (iii) 38‐week‐old mice that had an ovariectomy or sham operation early in life; (iv) 38‐week‐old mice that had been treated with progesterone or vehicle containing implants from 8 to 36 weeks. Transcript profiling was carried out using Affymetrix Gene ST 1.1 arrays, and data were normalized. We identified 60 differentially regulated transcripts associated with advancing age (group 1). We validated these changes in group 2 (*P* for overlap = 5.8 × 10^−46^). Early ovariectomy prevented the age‐related changes in myometrial transcript profile. Similarly, progesterone‐mediated long‐term ovarian suppression prevented the age‐related changes in myometrial transcript profile. Interferon regulatory factor 7 (Irf7) mRNA was regulated by age and hormonal exposure, and was identified as a predicted regulator of the other differentially expressed transcripts by both promoter sequence and canonical pathway activation analysis (*P *= 8.47 × 10^−5^ and *P *< 10^−10^, respectively). Immunohistochemistry demonstrated IRF7 in both mouse and human myometrium. We conclude the following: (i) Myometrial aging in mice is associated with reproducible changes in transcript profile; (ii) these changes can be prevented by interventions which inhibit cyclical changes in the female sex hormones; and (iii) IRF7 may be an important regulator of myometrial function and aging.

## Introduction results and discussion

Reproduction is a key goal for all organisms and fertility declines with age. The relationship between aging in general and ‘reproductive aging’ has been well studied particularly in the gonads, with relatively little attention paid to other portions of the female reproductive tract (Meldrum, 2013). There has been a marked increase in the average age at first childbirth, and the rates of cesarean delivery have risen (Ecker & Frigoletto, 2007; Smith *et al*., 2008). We observed that the contractile properties of isolated strips of human uterine smooth muscle deteriorated with increasing maternal age (Smith *et al*., 2008) and hypothesized that the increased risk of cesarean delivery in older mothers may be due to an adverse effect of aging on uterine contraction.

Delaying first pregnancy generally results in prolonged exposure to cyclical changes in circulating estrogen and progesterone, either endogenous (among women using abstinence or an intrauterine contraceptive device) or exogenous (combined oral contraceptive pill). Prolonged prepregnancy exposure to cyclical stimulation by the female sex hormones is a manifestation of modern societal and contraceptive developments which are unphysiological when considered in an evolutionary perspective. It is likely that exposure to ovarian hormones would have been characterized by late menarche, short duration exposure to the ovarian cycle followed by pregnancy and prolonged lactation, with the sequence being repeated after lactation (Eaton *et al*., 1994). We hypothesized that the adverse effect of increasing maternal age at the time of a first birth was related to prolonged exposure of the uterus to cyclical stimulation by the female sex hormones. Consistent with this view, we demonstrated that an increased menarche to first birth interval was associated with increased risk of operative first delivery (Smith, 2009; Smith & Froen, 2015). Despite the consistency of these data, the above hypothesis is only testable experimentally in animals. The aims of the present study were to determine whether any effect of aging was modifiable by manipulation of the exposure to the female sex hormones and to identify regulators of transcription that might be involved in the process.

**Figure 1:**
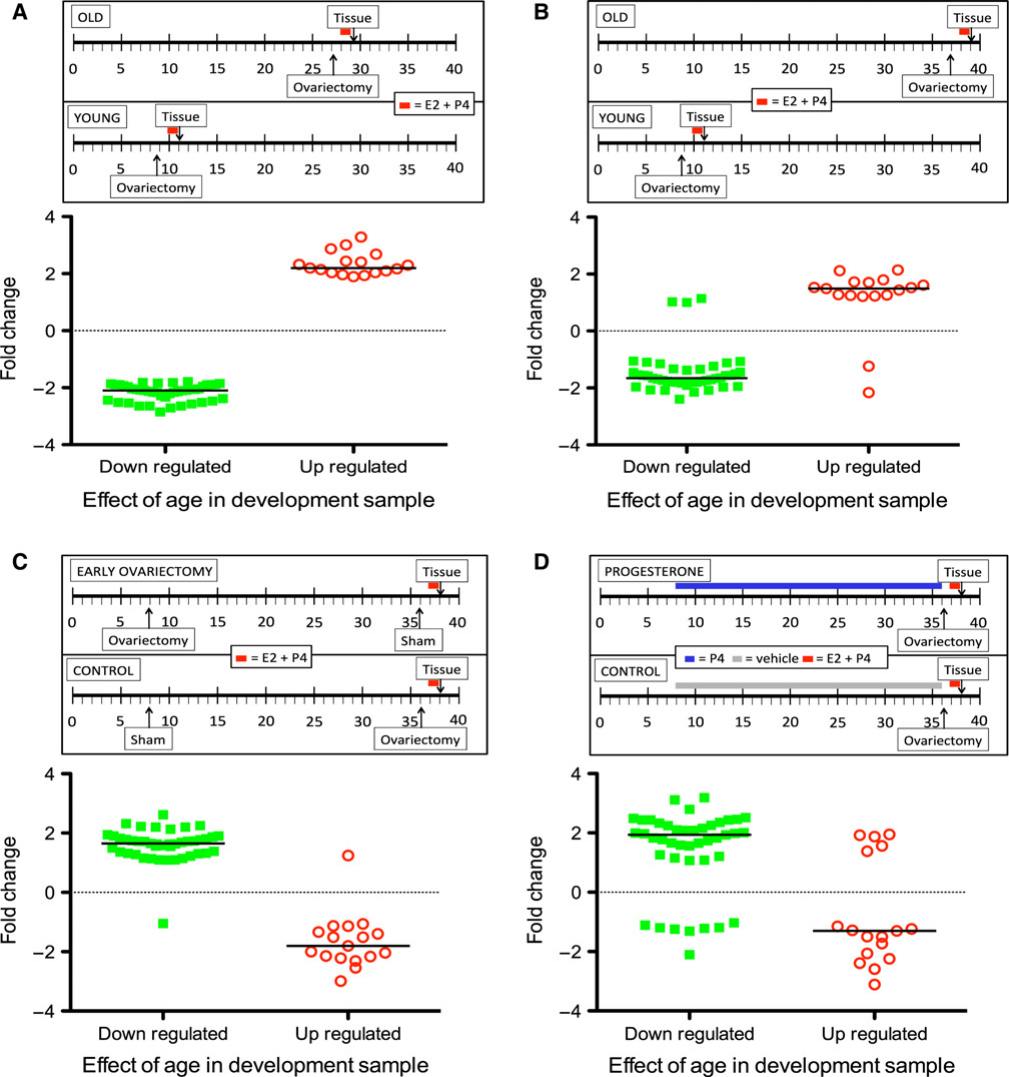
Time lines (scale in weeks) for study of aging and hormonal manipulation in mice and analysis of genes differentially expressed with advancing age. (A) Results from analysis of development sample. Panels B‐D are the fold changes in the same genes in a series of different experiments. (B)Validation sample of older versus young. (C) Older animals that had an ovariectomy at ~8 weeks of life vs older sham‐operated animals. (D) Older animals treated with progesterone (8 to 36–38 weeks) vs older animals receiving vehicle. In all four panels, green squares represent transcripts which were downregulated and red circles transcripts which were upregulated in older animals in the development sample. In all cases, the mean fold change was statistically significant different from 1 (1 sample t‐test with prior transformation to a normal distribution if required; all *P *< 0.001, except genes upregulated in older animals, comparing progesterone and vehicle where *P *= 0.03). Horizontal bars are the median fold change. Tissue = Time of culling and collection of myometrium. E2 + P4 =  Administration of estrogen and progesterone. P4 =  Animal treated with progesterone implant. Details of hormone manipulation are provided in Supporting Information.

We compared the myometrial transcript profile from a cohort of young and older mice (group 1, 10‐ to 12‐week‐ vs 28‐ to 30‐week‐old animals) and using the intersection of 2 distinct statistical methods (described in the supplementary Information) identified 60 differentially expressed transcripts. We validated these changes in a second cohort of young and older animals (group 2, 10‐ to 12‐week‐ vs 38‐ to 40‐week‐old mice). We next determined whether the age‐related changes in transcript profile could be prevented by blocking ovarian cyclicity. We compared older animals (38–40 weeks), which had an ovariectomy in early life with sham‐operated controls (group 3). Transcripts downregulated by age were upregulated in older animals that had an early ovariectomy. The converse was observed for genes which had been upregulated by age. We studied a second cohort of older animals in which ovarian cyclicity had been blocked by a different method, namely prolonged, continuous treatment with progesterone (group 4). Again, the transcripts which had been downregulated by age were upregulated in older animals by prolonged exposure to progesterone compared with vehicle treated controls. The converse was observed for genes that had been upregulated by age (Figs 1A–D and S1; Tables S1–S5).

**Figure 2:**
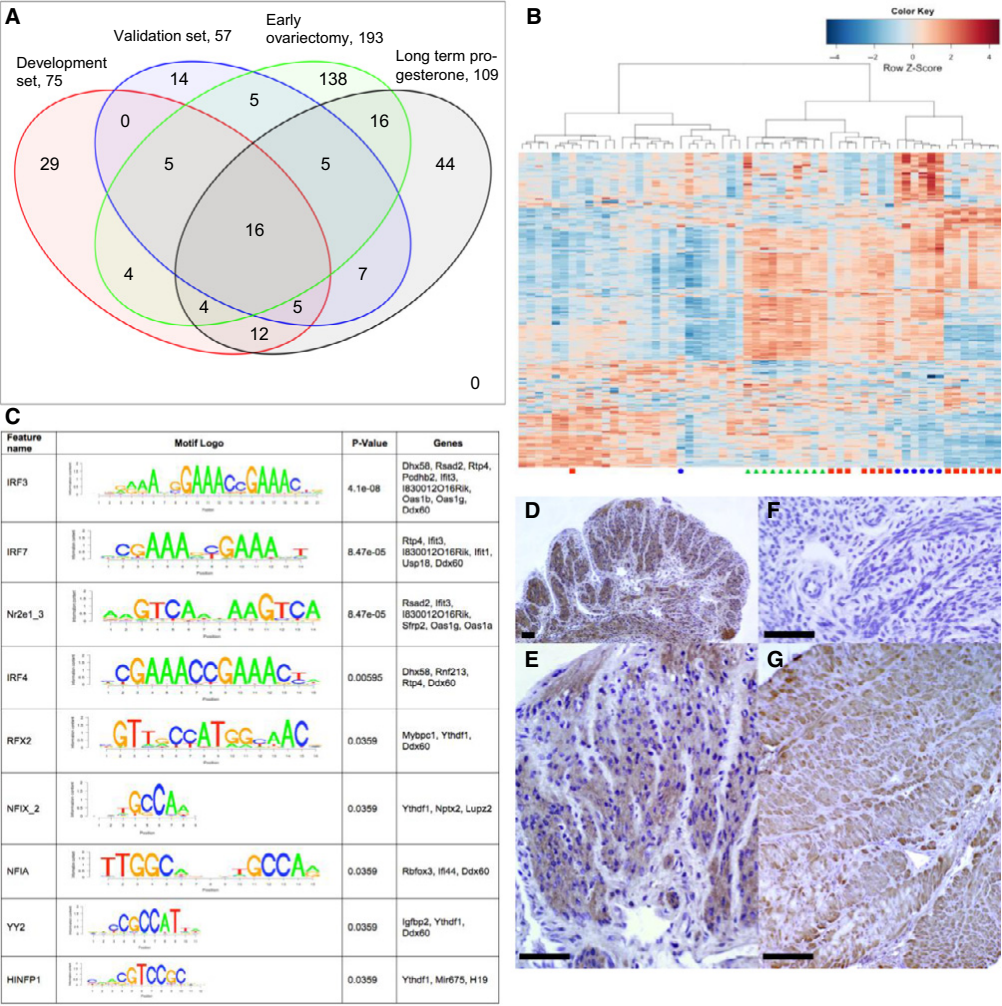
(A) Venn diagram indicating the overlaps among the differentially regulated transcripts from the 4 experimental groups. The number of regulated transcripts is given for each group. (B) Hierarchical clustering based on the 304 regulated transcripts. Red squares indicate young animals, blue circles older animals that had ovariectomy in early life, and green triangles older animals treated with long‐term progesterone. Columns without symbols indicate older animals that received sham, vehicle, or no treatment. (C) Motifs of transcription factor‐binding sites overrepresented in the 5 kb upstream region of the differentially regulated transcripts from the 10‐ to 12‐ vs 28‐ to 30‐week myometrium. (D and E) Uterus form 10‐week‐old mice immunostained with anti‐IRF7 antibody or (F) with the same antibody pre‐absorbed with an excess of the peptide against which it was raised. (G) Human myometrium stained under the same conditions. Brown indicates positive staining. Scale bar is 100 μm.

There was considerable overlap between the lists of differentially expressed transcripts (Fig. 2A), and this was highly statistically significant (all *P *< 10^−55^ Table S18). Hierarchical clustering based on the 304 transcripts differentially regulated in any of the 4 experimental groups (Tables S1–S4) showed that 30 of 32 animals exposed to fewer estrus cycles (i.e., young animals and older animals which had early ovariectomy or had been treated with progesterone) fell in 1 major branch. Similarly, 25 of 26 animals exposed to more estrus cycles (older animals which did not receive experimental intervention) fell in the other major branch (Fig. 2B). Thus, in the older animals in which ovarian cyclicity had been blocked, the myometrial transcript profile was similar to that of young animals.

Transcripts associated with the interferon response are significantly regulated (Tables S6–S13). Furthermore, 2 distinct analytical approaches (IPA, literature based) and motif enrichment (DNA sequence based) both highlighted members of the IRF (interferon regulatory factor, IRF1, IRF3, IRF7,) family of transcription factors and STAT1 and STAT2 as candidate transcriptional regulators of the changes observed (Tables S14–S17). Moreover, the transcript encoding IRF7 itself was significantly regulated in all four groups (Table S19). The IRFs, and particularly IRF7, are central to the host antiviral response (Honda *et al*., 2005), but recently other roles have been identified, particularly in mediating cardiac hypertrophy (Jiang *et al*., 2014). This and related work suggests that IRF7 is able to directly interact with cytosolic proteins such as IKKB and IRF9 interacts with PPAR (Wang *et al*., 2013). We examined the DNA 5 kb upstream of the transcription start sites of the aging‐regulated transcripts to identify transcription factor‐binding motifs. Three of the 9 significantly overrepresented motifs were for interferon regulatory factors (IRFs 3, 4, and 7, Fig. 2C). Furthermore, there is strong staining for IRF7 in mouse and human myometrium (Fig. 2D–G). Collectively, these data suggest that downregulation of IRF7 may have an important role in mediating the effect of prolonged cyclical stimulation by estrogen and progesterone on the uterus. These findings are, to our knowledge, the first indicating that production of IRF7 in myometrial cells controls any aspect of uterine function. However, our findings are consistent with previous studies that have shown that aging was associated with impaired upregulation of IRF7 in immune cells and that Irf7 mRNA can be regulated by estradiol replacement in the frontal cortex of middle‐aged rats (Stout‐Delgado *et al*., 2008; Sárvári *et al*., 2010).

In summary, we found experimental evidence to support our hypothesis that the effect of aging on the uterus is explained, at least in part, by the prior pattern of hormonal exposure. Hence, this aspect of uterine aging is clinically important as it suggests that the method of contraception used to delay first pregnancy may affect its outcome. Although we have focused on the muscle of the uterus and the risk of operative delivery, other estrogen and progesterone responsive tissues, both intrauterine and extrauterine, may exhibit similar properties.

## Funding information

This work was supported by the NIHR Cambridge Comprehensive Biomedical Research Centre, Addenbrooke's Charitable Trust, and the Evelyn Trust.

## Conflict of interest

The authors have nothing to disclose.

## Author contributions

DSC‐J and GCSS conceived experiments and analyzed data. HPC, YC, and YSR carried out experiments. HPC, SR, and BL analyzed data. All authors were involved in writing the manuscript and approved the final version.

## Supporting information


**Data S1** Methods and Results.
**Fig. S1** Analysis of transcripts differentially regulated with advanced age in validation and experimental groups.
**Tables S1–5** Differentially regulated transcripts.
**Tables S6–13** Molecular and cellular functions and canonical pathway analyses.
**Tables S14–17** Predicted upstream regulators.
**Table S18** Commonly identified transcript cluster IDs.
**Table S19** Fold change of Interferon Regulatory Factor 7 (IRF7).
