# Supplementary material for: Age‐related changes in murine myometrial transcript profile are mediated by exposure to the female sex hormones
Source: Aging Cell. 2015 Oct 21;15(1):177–80. doi: 10.1111/acel.12406 (PMC4717263; doi:10.1111/acel.12406)
Supplement: Supplementary file 1 — Data S1 Methods and Results. Fig. S1 Analysis of transcripts differentially regulated with advanced age in validation and experimental groups. Tables S1–5 Differentially regulated transcripts. Tables S6–13 Molecular and cellular functions and canonical pathway analyses. Tables S14–17 Predicted upstream regulators. Table S18 Commonly identified transcript cluster IDs. Table S19 Fold change of Interferon Regulatory Factor 7 (IRF7). [file ACEL-15-177-s001.docx]

**Supplementary Methods and Results.**

**Chong et al, Age-related changes in murine myometrial transcript profile are mediated by exposure to the female sex hormones.**

**EXPERIMENTAL PROCEDURES**

**Animal work**

Animals used were virgin female mice (C57BL/6J) and all experiments were carried out in accordance with the United Kingdom Animals Scientific Procedures Act 1986 which mandates ethical review. Animals had free access to food and water and were housed with constant temperature and humidity with a 12 hour light/dark cycle. Within each cohort, animals were weighed, ranked by descending order of body weight, and consecutive pairs selected to be intervention or control animals. All tissues were obtained from animals that had undergone ovariectomy (variable timing according to the experiment – see below). All animals were sacrificed following a standardized course of estrogen and progesterone which mimics the estrus cycle (Cheng et al. 2007). Estradiol was administered (25ng/g body weight) 7 days, 48 hours and 24 hours prior to sacrifice. Estradiol (0.5ng/g bodyweight) and progesterone (125ng/g body weight) were administered 6, 5, 4 and 3 days prior to sacrifice. Hormones were dissolved in arachis oil and injected subcutaneously.

In the initial analysis of the effect of age, young animals were sacrificed at 10-12 weeks of age and old animals at 28-30 weeks of age (Figure 1A, n=14). In these experiments, animals were kept in open ventilated cages. Subsequent experiments involved animals kept in individually ventilated cages. A second experiment was performed comparing young and old animals, with the aim of validating observed changes. However, a wider age difference was studied. Young animals still underwent sacrifice at 10-12 weeks of age, whereas older animals were sacrificed at 38-40 weeks (Figure 1B, n=15). The aim of using animals aged 38-40 weeks was to allow subsequent experiments to be performed in which older animals were exposed to prolonged manipulations of the sex hormones. Otherwise, the procedures were the same as the initial assessment of the effect of age.

**Early ovariectomy.**

To determine the contribution of prolonged exposure to the estrous cycle in causing age related changes, mice were randomized to ovariectomy (as described above) or a sham operation at 8-10 weeks of age (Figure 1C, n=14). The sham operation consisted of the same method of anaesthesia and a dorsolateral incision, but the ovary was exteriorized and then replaced within the abdominal cavity. At 36-38 weeks of age, animals were weighed and paired in descending order of body weight. The reciprocal operation was performed, i.e. animals that had an ovariectomy at 8 weeks had a sham operation and animals that had a sham operation at 8 weeks had an ovariectomy. All procedures were otherwise as described above.

**Prolonged progesterone exposure.**

To determine the effect of blocking ovarian cyclicity by prolonged exposure to progesterone on age related changes, mice were randomized to receive implants containing progesterone or vehicle (Figure 1D, n=15) (Milligan & Cohen 1994). Twenty millimetre lengths of Silastic^TM^ tubing were employed (Dow Corning, Michigan, USA; inner diameter 1.57mm, outer diameter 3.18mm). The tubes were plugged 5mm from each end giving an “effective length” of 10mm. This length was chosen in order to deliver sufficient progesterone to cause ovarian suppression (Telfer et al. 1991). Progesterone was dissolved in arachis oil at a concentration of 1g/mL. For the control animals, implants were filled with arachis oil. All implants were sterilized by irradiation and incubated in 0.1% gelatin in phosphate buffered saline for 24 hours prior to use. Implants were inserted under isoflurane general anaesthesia at 8 weeks of age using a 5mm trocar. Following insertion, the wound was closed using autoclips which were removed seven days later. Implants were replaced every 21 days as previously described (Milligan & Cohen 1994). At 36-38 weeks, the implant was removed, ovariectomy was performed, animals were treated with estrogen and progesterone and sacrificed 15 days after ovariectomy, as described above.

Ovarian cyclicity (or otherwise) was determined by performing vaginal smears in progesterone treated and vehicle control mice. Vaginal smears were performed for 7 consecutive days at 23 weeks of age, and for 1 day at 30 and 36 weeks of age. Smears were graded according to the estrous stage, as previously described (Mettus & Rane 2003). Animals were deemed to be cycling normally if estrous occurred on at least one of the seven consecutive days, or if the cytology indicated proestrous, estrous or metestrous on one occasion at 30 or 36 weeks of age. A mouse that had persistently diestrous smears was considered to be acyclic. All progesterone treated mice were acyclic and all comparisons with vehicle treated mice were with animals where ovarian cycling was confirmed.

**RNA extraction and gene array analysis**

Animals were killed by cervical dislocation and the uterus incubated in RNAlater (Ambion, Paisley, UK) for 24 hours at 4°C. The endometrium was removed by sharp dissection and the myometrium frozen in liquid nitrogen and stored at -80°C prior to RNA extraction. Myometrial RNA was isolated and its quality determined as described in the Supplemental Information. Labelling and hybridization to Affymetrix Mouse Gene ST 1.1 arrays was carried out by the Genomics Core Lab of the NIHR Cambridge Comprehensive Biomedical Research Centre according to the manufacturer's instructions. In all analyses, intervention and control animals were analysed on the same plate. The Raw and normalized array data described here have been up-loaded to Gene Expression Omnibus (accession number GSE49150).

**Analysis of array data**

Normalization of microarray data was performed using the Bioconducter ‘oligo’ package run in R (version 2.14.1) (Carvalho & Irizarry 2010). The Robust Multichip Average (RMA) function was used and the probes summarized to the “core” set. Microarray data were all normalized together. Comparison of array data from pairs of experimental groups in the arrays was performed using CyberT and RankProducts, as previously described (Cordeaux et al. 2010; Belteki et al. 2010). The criteria used to define the threshold of significance were: P<0.001; posterior probability of differential expression (ppde)>0.99 and P<0.001; percent false positive (pfp)<0.01, and an absolute fold change of at least 1.4. Affymetrix probe IDs were annotated using the Affymetrix annotation downloaded from the Netaffx web site (www.affymetrix.com/analysis/index.affx MoGene-1_1-st-v1.na32.mm9.transcript.csv). Overlap between the lists of differentially regulated transcripts obtained from the separate experiments was determined using Microsoft Excel. The Venn diagram was prepared using vennCounts and vennDiagram in R. The probability of identifying a given degree of overlap was determined using the hypergeometric distribution function dhyper in R. Hierarchical clustering was performed with “heatplot” using Ward’s method also in R.

Some of the differentially expressed transcripts did not have a corresponding MGI (Mouse Genome Informatics Database) symbol and are labelled as “transcribed sequence” in the tables. Affymetrix probe sets that detect these were originally designed in 2007 to hybridize to intronic sequences, exonic sequences (used to normalize signals) and intra-genic sequences and at the time of these probes were designated as controls. However, since then, it has become apparent that regions of the genome thought to be silent are in fact transcribed. The precise fraction of the genome transcribed and the functional importance of such transcripts remains to be determined (Clark et al. 2011). Although many non-coding and other short RNAs have been included in the updated annotation, the annotation is incomplete. These probe sets may therefore be detecting as-yet poorly characterized transcripts, hence, we have included them in tables. The array platform also includes 45 true negative controls, i.e. 15mers with no match in the genome which were extended by the addition of 5 random base pairs at each end to give a range of different GC contents. None of these true negative control probe sets were found to differ significantly in any of the comparisons of array data.

The array platform employed contained multiple probes for each gene. For most well annotated genes, signals from multiple probes are summarized as one value and assigned to a single transcript cluster. However, where the data used to generate the Meta ProbeSet file (MPS, Affymetrix) is weaker, a single gene may be reported as multiple transcript clusters. All tables of differentially expressed clusters include all of these duplicates for completeness. However, in the graphical representation of data and the statistical comparison of the degree of overlap between array experiments, we collapsed multiple occurrences of unique gene names to a single occurrence to avoid a single gene with multiple transcript clusters unduly influencing the analysis. However, in practice, the pattern and statistical significance of analyses was minimally affected by whether we collapsed the results of these duplicates or treated all transcript clusters separately.

**Ingenuity Pathway Analysis, IPA**

Enrichment of molecular and cellular functions and canonical pathways in the set of differentially regulated transcripts was performed using Ingenuity Pathway Analysis (IPA) software (Ingenuity Systems, Redwood City, California, build version 140500, content version 12710793). IPA also identifies potential upstream transcriptional regulators (TR) by comparing the direction of fold change of the transcripts in the uploaded data set against published literature. The threshold for significance was set at p<0.05 (Fisher’s exact test with multiple testing correction using the algorithm defined by Benjamini and Hochberg (Benjamini & Hochberg 1995).

**Transcription factor binding site enrichment**

To investigate the possible enrichment of transcription factor binding sites in the differentially regulated transcripts identified in the initial comparison of the 8-10 vs 28-30 week old mice we used the “Motif Enrichment Tool” as described by Blatti and Sinah (Blatti & Sinha 2014) http://veda.cs.uiuc.edu/cgi-bin/MET/interface.pl. This examines the enrichment of transcription factor binding motifs (obtained from the high throughput SELEX TF motifs data set (Jolma et al. 2013)). We examined the 5kb upstream of the transcription start site with normalization for GC content. The target set size was 500.

**RNA extraction and gene array analysis**

All animals were killed by cervical dislocation and the uterus incubated in RNAlater (Ambion, Paisley, UK) for 24 hours at 4°C. The endometrium was removed by sharp dissection and the myometrium flash frozen in liquid nitrogen and stored at -80°C prior to RNA extraction. Myometrial samples were individually homogenized in MagMAX™ lysing binding solution concentrate (Applied Biosystems, Warrington, UK) containing β-mercaptoethanol using a bead beater for 20 seconds at 6m/s (FastPrep24 tube holder, MP Biomedicals, Cambridge, UK). After addition of isopropanol and mixing, the lysate was loaded into a 96-well plate and RNA extracted using the MagMAX™ Express Magnetic Particle Processor (Applied Biosystems) and RNA purity and quality was assessed using ultraviolet spectrophotometry and the Agilent 2100 bioanalyzer (Agilent Technologies UK, Wokingham, UK), using the RNA integrity number (RIN) (Schroeder et al. 2006). RNA samples containing genomic DNA were treated with DNase (Turbo DNA-free, Applied Biosystems) and reanalysed. Whole genome array was performed by the Genomics Core Lab of the NIHR Cambridge Comprehensive Biomedical Research Centre. The Affymetrix Mouse Gene ST 1.1 arrays were used (Affymetrix UK, High Wycombe, UK). Labelling, hybridization and scanning was performed according to the manufacturer's instructions. The mean (SEM) RIN of the mouse RNA samples was 8.0 (0.13). In all analyses of mice, intervention and control animals were analysed on the same plate.

**Immunohistochemistry**

Human myometrial samples were obtained from non-laboring patients undergoing routine elective cesarean section, at 38 – 40 wk pregnancy. The study was approved by the Cambridgeshire 2 Research Ethics Committee and all patients gave their informed, written consent to participate. Indications for cesarean section included breech presentation and prior cesarean section but excluded multiple gestation.

Formalin fixed, paraffin wax embedded mouse or human uterine sections were de-waxed and rehydrated with xylene and a graded ethanol series (100%, 95%, 70%). Endogenous peroxidase was quenched (3% hydrogen peroxide in methanol, 10 min) followed by washing twice with phosphate buffered saline (PBS). Blocking was in 20% goat serum (GS, G9023 Sigma Aldrich, Dorset, UK) in PBS. A Rabbit polyclonal anti-IRF7 antibody (ab62505, Abcam, Cambridge, UK) was diluted to 5 μg/ml in 5% GS-PBS and incubated with the sections overnight at 4°C in a humidified chamber. Sections were washed (2 times 10 min in PBS) and incubated for 1 hour at room temperature with secondary antibody: biotinylated goat anti-rabbit IgG (E0432- DAKO, Cambridgeshire, UK) diluted to 1:400 in PBS. After three 10 min washes in PBS slides were incubated in Avidin/Biotin Complex (VECTASTAIN® Elite ABC kit 6100, Vector labs, Peterborough, UK) for 30 min at room temperature and washed (3 times 5 min in PBS). Following the development of the color reaction in 3,3’-diaminobenzidine (DAB, D4293, Sigma Aldrich, Dorset, UK) at approximately 3 min, the reaction was immediately stopped by immersing in tap water. Slides were counterstained in 50% haematoxylin and dehydrated in a graded ethanol series and xylene. Stained slides were cover-slipped using DePeX mountant.

Controls used were: rabbit IgG (final concentration 5µg/ml in 5% GS-PBS), solid phase absorbed (X0936, DAKO, Cambridgeshire, UK). Peptide pre-adsorbed antibody: The anti-IRF7 primary antibody was incubated overnight at 4°C with a 100 fold molar excess of a peptide corresponding to the epitope recognized by the antibody prior to use, (ab92594, Abcam, Cambridge, UK).

**Figure S1. Analysis of transcripts differentially regulated with advanced age in validation and experimental groups**. All panels show the fold changes of the transcripts that were significantly regulated in the development sample (timeline Figure 1A & Table S1). Panels A-C show the fold changes in the same genes in a series of different experiments: **A.** the validation samples in old versus young (timeline in Figure 1B). **B.** Old animals that had an early ovariectomy (at ~8 weeks of life) with old animals that had a sham operation at ~8 weeks (timeline Figure 1C). **C.** Old animals treated with progesterone from 8 to 36-38 weeks with old animals receiving vehicle from 8 to 36-38 weeks (timeline Figure 1D).





**Table S1 Differentially regulated transcripts in the 10-12 vs 28-30 week group.**

75 Transcript cluster IDs were differentially regulated by ageing to 28-30 weeks compared with 10-12 weeks. 67 transcript cluster IDs were recognized in IPA. The transcript cluster IDs are arranged in ascending order of the fold change. Several transcript cluster IDs existed for 3 transcripts: unc-13 homolog C (Unc13), ring finger 213 (Rnf213) and prune homolog 2 (Drosophila) (Prune 2). Fold changes are described as old/young. A negative fold change indicates down-regulation of the transcript cluster ID in the old group compared to the young group. *Transcript cluster IDs labeled N/A are transcribed sequences that have an as yet unknown gene name and symbol.

| Affymetrix Mouse Gene ST 1.1 ID | MGI Symbol | Gene Description | Fold Change Old (28-30)/ young (10-12) |
| --- | --- | --- | --- |
| 10502791 | Ifi44 | interferon-induced protein 44 | -2.85 |
| 10462623 | Ifit1 | interferon-induced protein with tetratricopeptide repeats 1 | -2.71 |
| 10462618 | Ifit3 | interferon-induced protein with tetratricopeptide repeats 3 | -2.65 |
| 10371660 | N/A | transcribed sequence | -2.64 |
| 10462621 | I830012O16Rik | RIKEN cDNA I830012O16 gene | -2.64 |
| 10541307 | Usp18 | ubiquitin specific peptidase 18 | -2.57 |
| 10339282 | N/A | transcribed sequence | -2.54 |
| 10350173 | Tnnt2 | troponin T2, cardiac | -2.52 |
| 10339870 | N/A | transcribed sequence | -2.51 |
| 10402347 | Ifi27l2a | interferon, alpha-inducible protein 27 like 2A | -2.47 |
| 10434778 | Rtp4 | receptor transporter protein 4 | -2.43 |
| 10496580 | Gbp3 | guanylate binding protein 3 | -2.38 |
| 10569102 | Irf7 | interferon regulatory factor 7 | -2.33 |
| 10524621 | Oasl2 | 2'-5' oligoadenylate synthetase-like 2 | -2.29 |
| 10437224 | Mx2 | myxovirus (influenza virus) resistance 2 | -2.21 |
| 10525158 | Oas1b | 2'-5' oligoadenylate synthetase 1B | -2.17 |
| 10391207 | Dhx58 | DEXH (Asp-Glu-X-His) box polypeptide 58 | -2.16 |
| 10343473 | N/A | transcribed sequence | -2.16 |
| 10553537 | Luzp2 | leucine zipper protein 2 | -2.14 |
| 10455961 | Iigp1 | interferon inducible GTPase 1 | -2.13 |
| 10569335 | H19 | H19 fetal liver mRNA | -2.10 |
| 10455954 | Gm4951 | predicted gene 4951 | -2.10 |
| 10371627 | Mybpc1 | myosin binding protein C, slow-type | -2.09 |
| 10533246 | Oas1g | 2'-5' oligoadenylate synthetase 1G | -2.09 |
| 10342620 | N/A | transcribed sequence | -2.07 |
| 10378068 | Xaf1 | XIAP associated factor 1 | -2.06 |
| 10455957 | N/A | cdna:genscan chromosome:NCBIM37:18:60196025:60406269:1 transcript_biotype:protein_coding | -2.06 |
| 10427222 | Amhr2 | anti-Mullerian hormone type 2 receptor | -2.04 |
| 10533256 | Oas1a | 2'-5' oligoadenylate synthetase 1A | -2.03 |
| 10594973 | Unc13c | unc-13 homolog C (C. elegans) | -2.02 |
| 10594948 | Unc13c | unc-13 homolog C (C. elegans) | -2.02 |
| 10383208 | Rnf213 | ring finger protein 213 | -1.99 |
| 10399710 | Rsad2 | radical S-adenosyl methionine domain containing 2 | -1.97 |
| 10526441 | Upk3b | uroplakin 3B | -1.95 |
| 10383206 | Rnf213 | ring finger protein 213 | -1.93 |
| 10562761 | Clec11a | C-type lectin domain family 11, member a | -1.90 |
| 10346191 | Stat1 | signal transducer and activator of transcription 1 | -1.89 |
| 10498119 | Frem2 | Fras1 related extracellular matrix protein 2 | -1.89 |
| 10362138 | Vnn1 | vanin 1 | -1.89 |
| 10345921 | 1500015O10Rik | RIKEN cDNA 1500015O10 gene | -1.88 |
| 10383210 | Rnf213 | ring finger protein 213 | -1.87 |
| 10599001 | Agtr2 | angiotensin II receptor, type 2 | -1.86 |
| 10564237 | Gm9801 | predicted gene 9801 | -1.85 |
| 10383233 | Rnf213 | ring finger protein 213 | -1.85 |
| 10383196 | Rnf213 | ring finger protein 213 | -1.84 |
| 10383152 | Rnf213 | ring finger protein 213 | -1.84 |
| 10571984 | Ddx60 | DEAD (Asp-Glu-Ala-Asp) box polypeptide 60 | -1.82 |
| 10383200 | Rnf213 | ring finger protein 213 | -1.82 |
| 10383214 | Rnf213 | ring finger protein 213 | -1.82 |
| 10383192 | Rnf213 | ring finger protein 213 | -1.82 |
| 10367224 | Stat2 | signal transducer and activator of transcription 2 | -1.81 |
| 10383204 | Rnf213 | ring finger protein 213 | -1.80 |
| 10383198 | Rnf213 | ring finger protein 213 | -1.79 |
| 10492798 | Sfrp2 | secreted frizzled-related protein 2 | -1.78 |
| 10594963 | Unc13c | unc-13 homolog C (C. elegans) | -1.78 |
| 10383202 | Rnf213 | ring finger protein 213 | -1.76 |
| 10383212 | Rnf213 | ring finger protein 213 | -1.75 |
| 10392415 | Rgs9 | regulator of G-protein signaling 9 | 1.89 |
| 10338690 | N/A | transcribed sequence | 1.94 |
| 10461878 | Prune2 | prune homolog 2 (Drosophila) | 1.96 |
| 10527332 | Nptx2 | neuronal pentraxin 2 | 1.97 |
| 10442786 | Tpsb2 | tryptase beta 2 | 2.04 |
| 10420247 | Mcpt4 | mast cell protease 4 | 2.09 |
| 10461869 | Prune2 | prune homolog 2 (Drosophila) | 2.12 |
| 10455050 | Pcdhb2 | protocadherin beta 2 | 2.14 |
| 10513739 | Tnc | tenascin C | 2.17 |
| 10418898 | Ppyr1 | pancreatic polypeptide receptor 1 | 2.20 |
| 10379727 | Gm11428 | predicted gene 11428 | 2.30 |
| 10393594 | D11Bwg0517e | DNA segment, Chr 11, Brigham | 2.32 |
| 10501104 | Slc6a17 | solute carrier family 6 (neurotransmitter transporter), member 17 | 2.41 |
| 10523231 | Art3 | ADP-ribosyltransferase 3 | 2.44 |
| 10347277 | Igfbp2 | insulin-like growth factor binding protein 2 | 2.69 |
| 10472136 | Galnt13 | UDP-N-acetyl-alpha-D-galactosamine:polypeptide N-acetylgalactosaminyltransferase 13 | 2.87 |
| 10580635 | Ces1d | carboxylesterase 1D | 3.01 |
| 10497451 | Cpa3 | carboxypeptidase A3, mast cell | 3.28 |

**Table S2 Differentially regulated transcripts in the 10-12 vs 38-40 week group.**

57 transcript cluster IDs were differentially regulated by ageing to 38-40 weeks compared with 10-12 weeks. 52 transcript cluster IDs were recognized in IPA. 10 transcript cluster IDs were duplicates of 2 transcripts: CD80 molecule and Ring finger 213 (Rnf213). Fold changes are described as old/young. A negative fold change indicates down-regulation of the probe set in the old group compared to the young group. Transcript cluster IDs labeled N/A are transcribed sequences that have an as yet unknown gene name and symbol.

| Affymetrix Mouse Gene ST 1.1 ID | MGI Symbol | Gene Description | FC Old (38-40)/ young (10-12) |
| --- | --- | --- | --- |
| 10338379 | N/A | transcribed sequence | -2.47 |
| 10462623 | Ifit1 | interferon-induced protein with tetratricopeptide repeats 1 | -2.40 |
| 10402347 | Ifi27l2a | interferon, alpha-inducible protein 27 like 2A | -2.15 |
| 10569102 | Irf7 | interferon regulatory factor 7 | -2.08 |
| 10502791 | Ifi44 | interferon-induced protein 44 | -2.08 |
| 10399710 | Rsad2 | radical S-adenosyl methionine domain containing 2 | -2.07 |
| 10526441 | Upk3b | uroplakin 3B | -1.97 |
| 10599001 | Agtr2 | angiotensin II receptor, type 2 | -1.95 |
| 10422164 | Ednrb | endothelin receptor type B | -1.92 |
| 10531415 | Cxcl10 | chemokine (C-X-C motif) ligand 10 | -1.92 |
| 10455954 | Gm4951 | predicted gene 4951 | -1.91 |
| 10385504 | Gm5431 | predicted gene 5431 | -1.90 |
| 10461594 | Ms4a4c | membrane-spanning 4-domains, subfamily A, member 4C | -1.88 |
| 10351880 | E430029J22Rik | RIKEN cDNA E430029J22 gene | -1.88 |
| 10566026 | Folr2 | folate receptor 2 (fetal) | -1.87 |
| 10537062 | Mest | mesoderm specific transcript | -1.87 |
| 10436623 | Chodl | chondrolectin | -1.85 |
| 10343029 | N/A | transcribed sequence | -1.84 |
| 10383208 | Rnf213 | ring finger protein 213 | -1.83 |
| 10541307 | Usp18 | ubiquitin specific peptidase 18 | -1.83 |
| 10351873 | Pyhin1 | pyrin and HIN domain family, member 1 | -1.82 |
| 10455961 | Iigp1 | interferon inducible GTPase 1 | -1.82 |
| 10462618 | Ifit3 | interferon-induced protein with tetratricopeptide repeats 3 | -1.81 |
| 10391207 | Dhx58 | DEXH (Asp-Glu-X-His) box polypeptide 58 | -1.80 |
| 10562761 | Clec11a | C-type lectin domain family 11, member a | -1.79 |
| 10383233 | Rnf213 | ring finger protein 213 | -1.78 |
| 10383198 | Rnf213 | ring finger protein 213 | -1.78 |
| 10434778 | Rtp4 | receptor transporter protein 4 | -1.77 |
| 10524621 | Oasl2 | 2'-5' oligoadenylate synthetase-like 2 | -1.76 |
| 10441233 | Mx1 | myxovirus (influenza virus) resistance 1 | -1.75 |
| 10571984 | Ddx60 | DEAD (Asp-Glu-Ala-Asp) box polypeptide 60 | -1.74 |
| 10420488 | D14Ertd668e | DNA segment, Chr 14, ERATO Doi 668, expressed | -1.74 |
| 10383200 | Rnf213 | ring finger protein 213 | -1.71 |
| 10531980 | Gbp9 | guanylate-binding protein 9 | -1.70 |
| 10383214 | Rnf213 | ring finger protein 213 | -1.70 |
| 10383192 | Rnf213 | ring finger protein 213 | -1.70 |
| 10571815 | Gpm6a | glycoprotein m6a | -1.67 |
| 10341814 | N/A | transcribed sequence | -1.67 |
| 10393594 | D11Bwg0517e | DNA segment, Chr 11, Brigham | 1.71 |
| 10355984 | Serpine2 | serine (or cysteine) peptidase inhibitor, clade E, member 2 | 1.75 |
| 10381809 | Itgb3 | integrin beta 3 | 1.75 |
| 10513739 | Tnc | tenascin C | 1.79 |
| 10435712 | Cd80 | CD80 antigen | 1.83 |
| 10598493 | Pcsk1n | proprotein convertase subtilisin/kexin type 1 inhibitor | 1.87 |
| 10453250 | N/A | transcribed sequence | 1.87 |
| 10435704 | Cd80 | CD80 antigen | 1.88 |
| 10413609 | Mustn1 | musculoskeletal, embryonic nuclear protein 1 | 1.98 |
| 10352798 | Kcnh1 | potassium voltage-gated channel, subfamily H (eag-related), member 1 | 2.11 |
| 10472136 | Galnt13 | UDP-N-acetyl-alpha-D-galactosamine:polypeptide N-acetylgalactosaminyltransferase 13 | 2.12 |
| 10523231 | Art3 | ADP-ribosyltransferase 3 | 2.14 |
| 10577782 | Htra4 | HtrA serine peptidase 4 | 2.18 |
| 10360415 | Grem2 | gremlin 2 homolog, cysteine knot superfamily (Xenopus laevis) | 2.19 |
| 10381798 | Myl4 | myosin, light polypeptide 4 | 2.20 |
| 10508272 | Csmd2 | CUB and Sushi multiple domains 2 | 2.23 |
| 10553967 | Pcsk6 | proprotein convertase subtilisin/kexin type 6 | 2.32 |
| 10342035 | N/A | transcribed sequence | 2.48 |
| 10355813 | Ptprn | protein tyrosine phosphatase, receptor type, N | 2.61 |

**Table S3 Differentially regulated transcripts following ovarian suppression by ovariectomy at 10-12 weeks.**

193 transcript cluster IDs were differentially regulated in the by early compared with late ovariectomy. 140 transcript cluster IDs were recognized in IPA. 19 transcript cluster IDs were replicates of 8 transcripts: immunoglobulin kappa chain variable 28 (V28)(Igk-V28), Ring finger 213 (RNF 213), Immunoglobulin heavy constant gamma 3(Ighg3), interferon activated gene 204 (Ifi204), signal-regulatory protein beta 1A (Sirpb1a), chemokine (C-C motif) receptor 5 (gene/pseudogene) (CCR5), chemokine (C-C motif) ligand 21 (CCL21) and T cell specific GTPase 1(Tgtp1/Tgtp2). 191 transcript cluster IDs were differentially regulated. Fold changes are described as late ovariectomy/early ovariectomy. A negative fold change indicates down-regulation of the probe set in the cycling group compared to the non-cycling group. Transcript cluster IDs labeled N/A are transcribed sequences that have an as yet unknown gene name and symbol.

| Affymetrix Mouse Gene ST 1.1 ID | MGI Symbol | Gene Description | Fold Change (Late Ovx/ Early Ovx) |
| --- | --- | --- | --- |
| 10538880 | Igk-V1 | immunoglobulin kappa chain variable 1 (V1) | -11.44 |
| 10403031 | V165-D-J-C mu | IgM variable region | -9.43 |
| 10545242 | Igk-V19-20 | immunoglobulin kappa chain variable 19 (V19)-20 | -8.66 |
| 10545180 | Gm10879 | predicted gene 10879 | -8.33 |
| 10403069 | Igh-6 | immunoglobulin heavy chain 6 (heavy chain of IgM) | -8.26 |
| 10545215 | Igk-V28 | immunoglobulin kappa chain variable 28 (V28) | -7.61 |
| 10538868 | N/A | Mus musculus mRNA for anti-human CD19 monoclonal antibody 4G7 immunoglobulin kappa light chain (IGK gene). | -6.45 |
| 10403048 | Ighv1-72 | immunoglobulin heavy variable V1-72 | -5.29 |
| 10531126 | Igj | immunoglobulin joining chain | -5.28 |
| 10538903 | Igk-V28 | immunoglobulin kappa chain variable 28 (V28) | -5.06 |
| 10403038 | LOC382693 | similar to immunoglobulin heavy chain | -4.96 |
| 10538924 | LOC100046496 | similar to Ig kappa V-region 24B | -4.86 |
| 10403043 | Ighv1-72 | immunoglobulin heavy variable V1-72 | -4.82 |
| 10403018 | IghmAC38.205.12 | Ig mu chain V region AC38 205.12 | -4.67 |
| 10403028 | LOC382693 | similar to immunoglobulin heavy chain | -4.56 |
| 10538882 | Gm5571 | predicted gene 5571 | -4.49 |
| 10544932 | Inmt | indolethylamine N-methyltransferase | -4.35 |
| 10403015 | AI324046 | expressed sequence AI324046 | -4.25 |
| 10403060 | Ighv1-72 | immunoglobulin heavy variable V1-72 | -4.07 |
| 10403021 | N/A | cdna:known chromosome:NCBIM37:12:116175324:116175617:-1 gene:ENSMUSG00000076707 gene_biotype:IG_V_gene transcript_biotype:IG_V_gene | -3.83 |
| 10545212 | Gm5574 | immunoglobulin kappa chain variable 12-47 | -3.82 |
| 10545220 | Gm16848 | predicted gene, 16848 | -3.78 |
| 10545198 | Igkv4-71 | immunoglobulin kappa chain variable 4-71 | -3.64 |
| 10545177 | N/A | Mus musculus anti-CEA 79 single chain Fv fragment mRNA, partial cds. | -3.51 |
| 10545187 | Gm1502 | predicted gene 1502 | -3.39 |
| 10403073 | Ighg | Immunoglobulin heavy chain (gamma polypeptide) | -3.16 |
| 10545175 | LOC672291 | similar to Ig kappa chain V-V region MOPC 173 | -3.13 |
| 10545196 | Gm1419 | predicted gene 1419 | -3.13 |
| 10438405 | Igl-V1 | immunoglobulin lambda chain, variable 1 | -3.02 |
| 10545194 | Rprl1 | ribonuclease P RNA-like 1 | -2.98 |
| 10403054 | LOC435333 | similar to monoclonal antibody heavy chain | -2.97 |
| 10538921 | N/A | Mus musculus mRNA for anti-leukotriene C4 monoclonal antibody immunoglobulin kappa light chain, complete cds. | -2.86 |
| 10545184 | Gm10880 | predicted gene 10880 | -2.85 |
| 10545210 | Gm1524 | predicted gene 1524 | -2.79 |
| 10545235 | N/A | Mus musculus (cell line C3H/F2-6) chromosome 6 anti-DNA antibody light chain mRNA. | -2.79 |
| 10403079 | LOC435333 | similar to monoclonal antibody heavy chain | -2.73 |
| 10438415 | Igl-V2 | immunoglobulin lambda chain, variable 2 | -2.72 |
| 10576235 | Dpep1 | dipeptidase 1 (renal) | -2.62 |
| 10502791 | Ifi44 | interferon-induced protein 44 | -2.62 |
| 10339190 | N/A | transcribed sequence | -2.61 |
| 10340485 | N/A | transcribed sequence | -2.57 |
| 10545247 | Igk-V19-14 | immunoglobulin kappa chain variable 19 (V19)-14 | -2.49 |
| 10545208 | Gm189 | predicted gene 189 | -2.46 |
| 10402864 | Ighg | Immunoglobulin heavy chain (gamma polypeptide) | -2.39 |
| 10545249 | N/A | cdna:known chromosome:NCBIM37:6:70407495:70407781:-1 gene:ENSMUSG00000076594 gene_biotype:IG_V_gene transcript_biotype:IG_V_gene | -2.38 |
| 10545190 | N/A | cdna:known chromosome:NCBIM37:6:69233788:69234313:-1 gene:ENSMUSG00000076548 gene_biotype:protein_coding transcript_biotype:protein_coding | -2.33 |
| 10562761 | Clec11a | C-type lectin domain family 11, member a | -2.32 |
| 10339081 | N/A | transcribed sequence | -2.31 |
| 10538871 | Gm4964 | predicted gene 4964 | -2.30 |
| 10576835 | Cd209f | CD209f antigen | -2.26 |
| 10462623 | Ifit1 | interferon-induced protein with tetratricopeptide repeats 1 | -2.25 |
| 10569102 | Irf7 | interferon regulatory factor 7 | -2.20 |
| 10350173 | Tnnt2 | troponin T2, cardiac | -2.20 |
| 10403034 | LOC100046275 | ig heavy chain V-II region SESS-like | -2.19 |
| 10591781 | Anln | anillin, actin binding protein | -2.17 |
| 10389207 | Ccl5 | chemokine (C-C motif) ligand 5 | -2.17 |
| 10344479 | N/A | transcribed sequence | -2.16 |
| 10360373 | E030037K03Rik | RIKEN cDNA E030037K03 gene | -2.15 |
| 10490150 | Zbp1 | Z-DNA binding protein 1 | -2.13 |
| 10399710 | Rsad2 | radical S-adenosyl methionine domain containing 2 | -2.13 |
| 10454709 | Kif20a | kinesin family member 20A | -2.13 |
| 10436623 | Chodl | chondrolectin | -2.12 |
| 10487823 | Siglec1 | sialic acid binding Ig-like lectin 1, sialoadhesin | -2.02 |
| 10351509 | Fcgr4 | Fc receptor, IgG, low affinity IV | -2.02 |
| 10603746 | Maob | monoamine oxidase B | -2.01 |
| 10379636 | Slfn4 | schlafen 4 | -2.00 |
| 10541564 | Clec4a3 | C-type lectin domain family 4, member a3 | -2.00 |
| 10466314 | N/A | cdna:genscan chromosome:NCBIM37:19:12603244:12604908:-1 transcript_biotype:protein_coding | -2.00 |
| 10512470 | Cd72 | CD72 antigen | -2.00 |
| 10497358 | Sirpb1b | signal-regulatory protein beta 1B | -1.99 |
| 10412126 | Il31ra | interleukin 31 receptor A | -1.98 |
| 10351873 | Pyhin1 | pyrin and HIN domain family, member 1 | -1.95 |
| 10462618 | Ifit3 | interferon-induced protein with tetratricopeptide repeats 3 | -1.94 |
| 10362186 | Moxd1 | monooxygenase, DBH-like 1 | -1.94 |
| 10385504 | Gm5431 | predicted gene 5431 | -1.91 |
| 10533198 | Oas2 | 2'-5' oligoadenylate synthetase 2 | -1.91 |
| 10552406 | Nkg7 | natural killer cell group 7 sequence | -1.91 |
| 10434778 | Rtp4 | receptor transporter protein 4 | -1.91 |
| 10523359 | Cxcl13 | chemokine (C-X-C motif) ligand 13 | -1.90 |
| 10391207 | Dhx58 | DEXH (Asp-Glu-X-His) box polypeptide 58 | -1.90 |
| 10389143 | Slfn8 | schlafen 8 | -1.89 |
| 10383208 | Rnf213 | ring finger protein 213 | -1.89 |
| 10516966 | BC013712 | cDNA sequence BC013712 | -1.88 |
| 10385518 | Tgtp1 | T-cell specific GTPase 1 | -1.88 |
| 10455954 | Gm4951 | predicted gene 4951 | -1.87 |
| 10542140 | Klrb1f | killer cell lectin-like receptor subfamily B member 1F | -1.87 |
| 10541555 | Clec4a1 | C-type lectin domain family 4, member a1 | -1.86 |
| 10342297 | N/A | transcribed sequence | -1.85 |
| 10340183 | N/A | transcribed sequence | -1.85 |
| 10461622 | Ms4a6b | membrane-spanning 4-domains, subfamily A, member 6B | -1.83 |
| 10455970 | BC023105 | cDNA sequence BC023105 | -1.83 |
| 10541307 | Usp18 | ubiquitin specific peptidase 18 | -1.82 |
| 10531415 | Cxcl10 | chemokine (C-X-C motif) ligand 10 | -1.81 |
| 10402347 | Ifi27l2a | interferon, alpha-inducible protein 27 like 2A | -1.81 |
| 10383212 | Rnf213 | ring finger protein 213 | -1.79 |
| 10512766 | Trim14 | tripartite motif-containing 14 | -1.79 |
| 10533246 | Oas1g | 2'-5' oligoadenylate synthetase 1G | -1.78 |
| 10599001 | Agtr2 | angiotensin II receptor, type 2 | -1.78 |
| 10588479 | Tlr9 | toll-like receptor 9 | -1.78 |
| 10385533 | Tgtp1 | T-cell specific GTPase 1 | -1.77 |
| 10406928 | Cd180 | CD180 antigen | -1.76 |
| 10394054 | Cd7 | CD7 antigen | -1.76 |
| 10383233 | Rnf213 | ring finger protein 213 | -1.76 |
| 10376324 | Gm12250 | predicted gene 12250 | -1.76 |
| 10566358 | Trim30a | tripartite motif-containing 30A | -1.75 |
| 10585276 | Pou2af1 | POU domain, class 2, associating factor 1 | -1.75 |
| 10455961 | Iigp1 | interferon inducible GTPase 1 | -1.75 |
| 10501063 | Cd53 | CD53 antigen | -1.74 |
| 10566583 | Gm8995 | predicted gene 8995 | -1.73 |
| 10524621 | Oasl2 | 2'-5' oligoadenylate synthetase-like 2 | -1.73 |
| 10398907 | Pld4 | phospholipase D family, member 4 | -1.73 |
| 10571984 | Ddx60 | DEAD (Asp-Glu-Ala-Asp) box polypeptide 60 | -1.72 |
| 10590438 | Fam198a | family with sequence similarity 198, member A | -1.72 |
| 10416181 | Stc1 | stanniocalcin 1 | -1.72 |
| 10392808 | Cd300ld | CD300 molecule-like family member d | -1.72 |
| 10339802 | N/A | transcribed sequence | -1.71 |
| 10497349 | Sirpb1a | signal-regulatory protein beta 1A | -1.71 |
| 10500335 | Fcgr1 | Fc receptor, IgG, high affinity I | -1.70 |
| 10590635 | Ccr5 | chemokine (C-C motif) receptor 5 | -1.70 |
| 10598013 | Ccr5 | chemokine (C-C motif) receptor 5 | -1.70 |
| 10608681 | N/A | transcribed sequence | -1.69 |
| 10404606 | Ly86 | lymphocyte antigen 86 | -1.69 |
| 10430344 | Il2rb | interleukin 2 receptor, beta chain | -1.69 |
| 10338650 | N/A | transcribed sequence | -1.69 |
| 10461605 | Ms4a4b | membrane-spanning 4-domains, subfamily A, member 4B | -1.69 |
| 10494271 | Ctss | cathepsin S | -1.68 |
| 10467578 | Pik3ap1 | phosphoinositide-3-kinase adaptor protein 1 | -1.68 |
| 10389231 | Ccl3 | chemokine (C-C motif) ligand 3 | -1.68 |
| 10541644 | Cd163 | CD163 antigen | -1.65 |
| 10531980 | Gbp9 | guanylate-binding protein 9 | -1.63 |
| 10502240 | Npnt | nephronectin | -1.62 |
| 10362201 | Ctgf | connective tissue growth factor | 1.45 |
| 10427035 | Nr4a1 | nuclear receptor subfamily 4, group A, member 1 | 1.49 |
| 10338550 | N/A | transcribed sequence | 1.52 |
| 10509238 | Htr1d | 5-hydroxytryptamine (serotonin) receptor 1D | 1.55 |
| 10338197 | N/A | transcribed sequence | 1.57 |
| 10342922 | N/A | transcribed sequence | 1.57 |
| 10343600 | N/A | transcribed sequence | 1.58 |
| 10416406 | Htr2a | 5-hydroxytryptamine (serotonin) receptor 2A | 1.58 |
| 10356305 | Htr2b | 5-hydroxytryptamine (serotonin) receptor 2B | 1.59 |
| 10493114 | Nes | nestin | 1.61 |
| 10351491 | Olfml2b | olfactomedin-like 2B | 1.61 |
| 10388194 | Spns2 | spinster homolog 2 (Drosophila) | 1.61 |
| 10536667 | Ptprz1 | protein tyrosine phosphatase, receptor type Z, polypeptide 1 | 1.62 |
| 10343681 | N/A | transcribed sequence | 1.63 |
| 10338942 | N/A | transcribed sequence | 1.63 |
| 10541753 | C530028O21Rik | RIKEN cDNA C530028O21 gene | 1.64 |
| 10455080 | Pcdhb9 | protocadherin beta 9 | 1.64 |
| 10353632 | Bai3 | brain-specific angiogenesis inhibitor 3 | 1.64 |
| 10482500 | Rnd3 | Rho family GTPase 3 | 1.65 |
| 10595324 | Htr1b | 5-hydroxytryptamine (serotonin) receptor 1B | 1.67 |
| 10381809 | Itgb3 | integrin beta 3 | 1.67 |
| 10343995 | N/A | transcribed sequence | 1.68 |
| 10599927 | Aff2 | AF4/FMR2 family, member 2 | 1.68 |
| 10567564 | Cdr2 | cerebellar degeneration-related 2 | 1.68 |
| 10455054 | Pcdhb3 | protocadherin beta 3 | 1.68 |
| 10403743 | Inhba | inhibin beta-A | 1.71 |
| 10413609 | Mustn1 | musculoskeletal, embryonic nuclear protein 1 | 1.71 |
| 10487797 | Adam33 | a disintegrin and metallopeptidase domain 33 | 1.72 |
| 10552708 | Kcnc3 | potassium voltage gated channel, Shaw-related subfamily, member 3 | 1.72 |
| 10341467 | N/A | transcribed sequence | 1.72 |
| 10340452 | N/A | transcribed sequence | 1.72 |
| 10420366 | Gjb6 | gap junction protein, beta 6 | 1.73 |
| 10420616 | Sgcg | sarcoglycan, gamma (dystrophin-associated glycoprotein) | 1.75 |
| 10504127 | Ccl21a | chemokine (C-C motif) ligand 21A (serine) | 1.75 |
| 10504154 | Ccl21a | chemokine (C-C motif) ligand 21A (serine) | 1.75 |
| 10504183 | Ccl21a | chemokine (C-C motif) ligand 21A (serine) | 1.75 |
| 10512377 | Ccl21a | chemokine (C-C motif) ligand 21A (serine) | 1.75 |
| 10409282 | Ror2 | receptor tyrosine kinase-like orphan receptor 2 | 1.75 |
| 10433887 | Pkp2 | plakophilin 2 | 1.75 |
| 10342344 | N/A | transcribed sequence | 1.76 |
| 10409579 | Cxcl14 | chemokine (C-X-C motif) ligand 14 | 1.76 |
| 10409222 | Shc3 | src homology 2 domain-containing transforming protein C3 | 1.76 |
| 10590031 | Itga9 | integrin alpha 9 | 1.78 |
| 10564482 | Synm | synemin, intermediate filament protein | 1.78 |
| 10497451 | Cpa3 | carboxypeptidase A3, mast cell | 1.81 |
| 10344096 | N/A | transcribed sequence | 1.91 |
| 10508272 | Csmd2 | CUB and Sushi multiple domains 2 | 1.93 |
| 10534667 | Serpine1 | serine (or cysteine) peptidase inhibitor, clade E, member 1 | 1.93 |
| 10501104 | Slc6a17 | solute carrier family 6 (neurotransmitter transporter), member 17 | 1.99 |
| 10455050 | Pcdhb2 | protocadherin beta 2 | 2.04 |
| 10436727 | ORF63 | open reading frame 63 | 2.06 |
| 10418898 | Ppyr1 | pancreatic polypeptide receptor 1 | 2.15 |
| 10513739 | Tnc | tenascin C | 2.17 |
| 10553967 | Pcsk6 | proprotein convertase subtilisin/kexin type 6 | 2.18 |
| 10393594 | D11Bwg0517e | DNA segment, Chr 11, Brigham | 2.21 |
| 10363921 | Pcdh15 | protocadherin 15 | 2.22 |
| 10607712 | Grpr | gastrin releasing peptide receptor | 2.25 |
| 10523231 | Art3 | ADP-ribosyltransferase 3 | 2.31 |
| 10381798 | Myl4 | myosin, light polypeptide 4 | 2.45 |
| 10490913 | Car3 | carbonic anhydrase 3 | 2.49 |
| 10472136 | Galnt13 | UDP-N-acetyl-alpha-D-galactosamine:polypeptide N-acetylgalactosaminyltransferase 13 | 2.54 |
| 10347277 | Igfbp2 | insulin-like growth factor binding protein 2 | 2.99 |

**Table S4 Differentially regulated transcripts associated with long-term progesterone treatment.**

109 transcript cluster IDs were differentially regulated by long-term progesterone treatment. 97 transcript cluster IDs were recognized in IPA. 27 transcript cluster IDs were duplicates of 6 transcripts: apolipoprotein L9b (Apol9a/Apol9b), guanylate binding protein 6 (Gbp6), GTPase, very large interferon inducible 1 (Gvin1), lymphocyte antigen 6 complex, locus A (Ly6a), Ring finger 213 (Rnf213) and T cell specific GTPase 1 (Tgtp1/Tgtp2). Fold changes are described as vehicle/P4. A negative fold change indicates down-regulation of the probe set in the vehicle (cycling) group compared to the P4 (non-cycling) group. Transcript cluster IDs labeled N/A are transcribed sequences that have an as yet unknown gene name and symbol.

| Affymetrix Mouse Gene ST 1.1 ID | MGI Symbol | Gene Description | Fold Change (Vehicle/P4) |
| --- | --- | --- | --- |
| 10379636 | Slfn4 | schlafen 4 | -3.93 |
| 10576835 | Cd209f | CD209f antigen | -3.22 |
| 10462623 | Ifit1 | interferon-induced protein with tetratricopeptide repeats 1 | -3.19 |
| 10425049 | Apol9b | apolipoprotein L 9b | -3.00 |
| 10379633 | Slfn1 | schlafen 1 | -2.85 |
| 10533256 | Oas1a | 2'-5' oligoadenylate synthetase 1A | -2.80 |
| 10343926 | N/A | transcribed sequence | -2.75 |
| 10430174 | Apol9a | apolipoprotein L 9a | -2.52 |
| 10569102 | Irf7 | interferon regulatory factor 7 | -2.51 |
| 10502791 | Ifi44 | interferon-induced protein 44 | -2.49 |
| 10533213 | Oas3 | 2'-5' oligoadenylate synthetase 3 | -2.49 |
| 10434778 | Rtp4 | receptor transporter protein 4 | -2.46 |
| 10490150 | Zbp1 | Z-DNA binding protein 1 | -2.46 |
| 10496580 | Gbp3 | guanylate binding protein 3 | -2.44 |
| 10462618 | Ifit3 | interferon-induced protein with tetratricopeptide repeats 3 | -2.44 |
| 10524631 | Oasl1 | 2'-5' oligoadenylate synthetase-like 1 | -2.40 |
| 10452316 | C3 | complement component 3 | -2.39 |
| 10399710 | Rsad2 | radical S-adenosyl methionine domain containing 2 | -2.36 |
| 10391207 | Dhx58 | DEXH (Asp-Glu-X-His) box polypeptide 58 | -2.33 |
| 10533246 | Oas1g | 2'-5' oligoadenylate synthetase 1G | -2.26 |
| 10525158 | Oas1b | 2'-5' oligoadenylate synthetase 1B | -2.24 |
| 10533198 | Oas2 | 2'-5' oligoadenylate synthetase 2 | -2.24 |
| 10541307 | Usp18 | ubiquitin specific peptidase 18 | -2.16 |
| 10383210 | Rnf213 | ring finger protein 213 | -2.13 |
| 10461594 | Ms4a4c | membrane-spanning 4-domains, subfamily A, member 4C | -2.12 |
| 10383212 | Rnf213 | ring finger protein 213 | -2.11 |
| 10571984 | Ddx60 | DEAD (Asp-Glu-Ala-Asp) box polypeptide 60 | -2.10 |
| 10441233 | Mx1 | myxovirus (influenza virus) resistance 1 | -2.10 |
| 10376324 | Gm12250 | predicted gene 12250 | -2.09 |
| 10383208 | Rnf213 | ring finger protein 213 | -2.09 |
| 10526441 | Upk3b | uroplakin 3B | -2.09 |
| 10455954 | Gm4951 | predicted gene 4951 | -2.08 |
| 10383152 | Rnf213 | ring finger protein 213 | -2.08 |
| 10383194 | Rnf213 | ring finger protein 213 | -2.07 |
| 10462613 | Ifit2 | interferon-induced protein with tetratricopeptide repeats 2 | -2.05 |
| 10542470 | Mgst1 | microsomal glutathione S-transferase 1 | -2.04 |
| 10538590 | Herc6 | hect domain and RLD 6 | -2.03 |
| 10500335 | Fcgr1 | Fc receptor, IgG, high affinity I | -2.03 |
| 10383214 | Rnf213 | ring finger protein 213 | -2.01 |
| 10455961 | Iigp1 | interferon inducible GTPase 1 | -2.01 |
| 10450374 | D17H6S56E-5 | DNA segment, Chr 17, human D6S56E 5 | -1.98 |
| 10524621 | Oasl2 | 2'-5' oligoadenylate synthetase-like 2 | -1.97 |
| 10383192 | Rnf213 | ring finger protein 213 | -1.97 |
| 10383202 | Rnf213 | ring finger protein 213 | -1.97 |
| 10420488 | D14Ertd668e | DNA segment, Chr 14, ERATO Doi 668, expressed | -1.97 |
| 10383206 | Rnf213 | ring finger protein 213 | -1.96 |
| 10385533 | Tgtp1 | T-cell specific GTPase 1 | -1.96 |
| 10383196 | Rnf213 | ring finger protein 213 | -1.95 |
| 10383233 | Rnf213 | ring finger protein 213 | -1.94 |
| 10385518 | Tgtp1 | T-cell specific GTPase 1 | -1.94 |
| 10378068 | Xaf1 | XIAP associated factor 1 | -1.94 |
| 10566026 | Folr2 | folate receptor 2 (fetal) | -1.93 |
| 10383204 | Rnf213 | ring finger protein 213 | -1.93 |
| 10531980 | Gbp9 | guanylate-binding protein 9 | -1.93 |
| 10385500 | Irgm1 | immunity-related GTPase family M member 1 | -1.91 |
| 10383200 | Rnf213 | ring finger protein 213 | -1.91 |
| 10338237 | N/A | transcribed sequence | -1.91 |
| 10389143 | Slfn8 | schlafen 8 | -1.90 |
| 10376326 | Irgm2 | immunity-related GTPase family M member 2 | -1.90 |
| 10531994 | Mpa2l | macrophage activation 2 like | -1.90 |
| 10487823 | Siglec1 | sialic acid binding Ig-like lectin 1, sialoadhesin | -1.89 |
| 10383198 | Rnf213 | ring finger protein 213 | -1.89 |
| 10566358 | Trim30a | tripartite motif-containing 30A | -1.89 |
| 10351509 | Fcgr4 | Fc receptor, IgG, low affinity IV | -1.88 |
| 10351873 | Pyhin1 | pyrin and HIN domain family, member 1 | -1.87 |
| 10458560 | Fgf1 | fibroblast growth factor 1 | -1.86 |
| 10461622 | Ms4a6b | membrane-spanning 4-domains, subfamily A, member 6B | -1.86 |
| 10395039 | Cmpk2 | cytidine monophosphate (UMP-CMP) kinase 2, mitochondrial | -1.84 |
| 10443195 | Hmga1 | high mobility group AT-hook 1 | -1.83 |
| 10367224 | Stat2 | signal transducer and activator of transcription 2 | -1.83 |
| 10473356 | Ube2l6 | ubiquitin-conjugating enzyme E2L 6 | -1.80 |
| 10566578 | Gm8979 | very large inducible GTPase 1 pseudogene | -1.80 |
| 10429564 | Ly6a | lymphocyte antigen 6 complex, locus A | -1.80 |
| 10588479 | Tlr9 | toll-like receptor 9 | -1.79 |
| 10531370 | Naaa | N-acylethanolamine acid amidase | -1.78 |
| 10444244 | Tap1 | transporter 1, ATP-binding cassette, sub-family B (MDR/TAP) | -1.78 |
| 10523012 | Dck | deoxycytidine kinase | -1.77 |
| 10566585 | Gm1966 | predicted gene 1966 | -1.77 |
| 10483110 | Ifih1 | interferon induced with helicase C domain 1 | -1.77 |
| 10383168 | Rnf213 | ring finger protein 213 | -1.76 |
| 10416566 | Epsti1 | epithelial stromal interaction 1 (breast) | -1.75 |
| 10338875 | N/A | transcribed sequence | -1.75 |
| 10429568 | Ly6c1 | lymphocyte antigen 6 complex, locus C1 | -1.74 |
| 10435457 | Parp9 | poly (ADP-ribose) polymerase family, member 9 | -1.74 |
| 10346191 | Stat1 | signal transducer and activator of transcription 1 | -1.74 |
| 10489569 | Pltp | phospholipid transfer protein | -1.73 |
| 10607705 | S100g | S100 calcium binding protein G | 2.05 |
| 10347277 | Igfbp2 | insulin-like growth factor binding protein 2 | 2.25 |
| 10553967 | Pcsk6 | proprotein convertase subtilisin/kexin type 6 | 2.33 |
| 10393594 | D11Bwg0517e | DNA segment, Chr 11, Brigham | 2.39 |
| 10577782 | Htra4 | HtrA serine peptidase 4 | 2.43 |
| 10338382 | N/A | transcribed sequence* | 2.50 |
| 10342539 | N/A | transcribed sequence | 2.56 |
| 10418898 | Ppyr1 | pancreatic polypeptide receptor 1 | 2.59 |
| 10511631 | Slc26a7 | solute carrier family 26, member 7 | 2.63 |
| 10343133 | N/A | transcribed sequence | 2.68 |
| 10352798 | Kcnh1 | potassium voltage-gated channel, subfamily H (eag-related), member 1 | 2.71 |
| 10420366 | Gjb6 | gap junction protein, beta 6 | 2.75 |
| 10508272 | Csmd2 | CUB and Sushi multiple domains 2 | 2.78 |
| 10441195 | Dscam | Down syndrome cell adhesion molecule | 3.01 |
| 10339199 | N/A | transcribed sequence | 3.04 |
| 10339116 | N/A | transcribed sequence | 3.05 |
| 10472136 | Galnt13 | UDP-N-acetyl-alpha-D-galactosamine:polypeptide N-acetylgalactosaminyltransferase 13 | 3.11 |
| 10355813 | Ptprn | protein tyrosine phosphatase, receptor type, N | 3.18 |
| 10338102 | N/A | transcribed sequence | 3.23 |
| 10340655 | N/A | transcribed sequence | 3.57 |
| 10607712 | Grpr | gastrin releasing peptide receptor | 3.80 |
| 10381798 | Myl4 | myosin, light polypeptide 4 | 4.19 |
| 10342604 | N/A | transcribed sequence | 5.79 |

**Table S5 Differentially regulated transcripts in the 10-12 vs 28-30 week group in the validation and experimental groups.**

60 unique transcript cluster IDs were differentially regulated by ageing to 28-30 weeks compared with 10-12 weeks and the fold changes in the validation and experimental groups are shown. The transcript cluster IDs are arranged in ascending order of the fold change. Fold changes are described as old/young. A negative fold change indicates down-regulation of the transcript cluster ID in the old group compared to the young group. *Transcript cluster IDs labeled N/A are transcribed sequences that have an as yet unknown gene name and symbol.

| **MGI Symbol** | **Gene Description** | **Fold Change old/young Development** | **Fold Change old/young Validation** | **Fold Change Early/late Ovex** | **Fold Change Progesterone/ vehicle** |
| --- | --- | --- | --- | --- | --- |
| Ifi44 | interferon-induced protein 44 | -2.85 | -2.08 | 2.62 | 2.49 |
| Ifit1 | interferon-induced protein with tetratricopeptide repeats 1 | -2.71 | -2.40 | 2.25 | 3.19 |
| Ifit3 | interferon-induced protein with tetratricopeptide repeats 3 | -2.64 | -1.81 | 1.94 | 2.44 |
| I830012O16Rik | RIKEN cDNA I830012O16 gene | -2.64 | -1.34 | 1.37 | -1.31 |
| 10371660 | #N/A | -2.64 | -1.96 | 1.65 | 2.44 |
| Usp18 | ubiquitin specific peptidase 18 | -2.57 | -1.83 | 1.82 | 2.16 |
| 10339282 | #N/A | -2.54 | 1.14 | 2.22 | -1.25 |
| Tnnt2 | troponin T2, cardiac | -2.52 | -1.58 | 2.20 | 1.98 |
| 10339870 | #N/A | -2.51 | -1.24 | 1.31 | -2.11 |
| Ifi27l2a | interferon, alpha-inducible protein 27 like 2A | -2.47 | -2.15 | 1.81 | 3.11 |
| Rtp4 | receptor transporter protein 4 | -2.43 | -1.77 | 1.91 | 2.46 |
| Gbp3 | guanylate binding protein 3 | -2.38 | -1.69 | 1.55 | 2.44 |
| Irf7 | interferon regulatory factor 7 | -2.33 | -2.08 | 2.20 | 2.51 |
| Oasl2 | 2'-5' oligoadenylate synthetase-like 2 | -2.29 | -1.76 | 1.73 | 1.97 |
| Mx2 | myxovirus (influenza virus) resistance 2 | -2.21 | -1.38 | 1.69 | 1.83 |
| Oas1b | 2'-5' oligoadenylate synthetase 1B | -2.17 | -1.66 | 1.63 | 2.24 |
| Dhx58 | DEXH (Asp-Glu-X-His) box polypeptide 58 | -2.16 | -1.80 | 1.90 | 2.33 |
| 10343473 | #N/A | -2.16 | 1.00 | 1.09 | -1.22 |
| Luzp2 | leucine zipper protein 2 | -2.14 | -1.15 | 1.32 | -1.03 |
| Iigp1 | interferon inducible GTPase 1 | -2.13 | -1.82 | 1.75 | 2.01 |
| H19 | H19 fetal liver mRNA | -2.10 | -1.73 | 1.50 | 1.26 |
| Gm4951 | predicted gene 4951 | -2.10 | -1.91 | 1.87 | 2.08 |
| Oas1g | 2'-5' oligoadenylate synthetase 1G | -2.09 | 1.02 | 1.10 | 1.16 |
| Mybpc1 | myosin binding protein C, slow-type | -2.09 | -1.71 | 1.78 | 2.26 |
| 10342620 | #N/A | -2.06 | -1.09 | -1.05 | 1.09 |
| 10455957 | #N/A | -2.06 | -1.51 | 1.56 | 1.94 |
| Xaf1 | XIAP associated factor 1 | -2.06 | -1.56 | 1.63 | 1.66 |
| Amhr2 | anti-Mullerian hormone type 2 receptor | -2.03 | -1.07 | 1.14 | 1.21 |
| Oas1a | 2'-5' oligoadenylate synthetase 1A | -2.03 | -1.55 | 1.39 | 2.80 |
| Rsad2 | radical S-adenosyl methionine domain containing 2 | -1.96 | -2.07 | 2.13 | 2.36 |
| Upk3b | uroplakin 3B | -1.95 | -1.97 | 1.28 | 2.09 |
| Unc13c | unc-13 homolog C (C. elegans) | -1.93 | -1.12 | 1.16 | -1.19 |
| Clec11a | C-type lectin domain family 11, member a | -1.90 | -1.79 | 2.32 | 1.57 |
| Frem2 | Fras1 related extracellular matrix protein 2 | -1.89 | -1.45 | 1.57 | 1.73 |
| Stat1 | signal transducer and activator of transcription 1 | -1.89 | -1.33 | 1.64 | 1.59 |
| Vnn1 | vanin 1 | -1.89 | -1.53 | 1.22 | -1.11 |
| 1500015O10Rik | RIKEN cDNA 1500015O10 gene | -1.88 | -1.45 | 1.15 | 1.66 |
| Agtr2 | angiotensin II receptor, type 2 | -1.86 | -1.95 | 1.78 | 1.78 |
| Gm9801 | predicted gene 9801 | -1.85 | -1.56 | 1.32 | 1.07 |
| Rnf213 | ring finger protein 213 | -1.83 | -1.64 | 1.72 | 1.99 |
| Ddx60 | DEAD (Asp-Glu-Ala-Asp) box polypeptide 60 | -1.82 | -1.74 | 1.72 | 2.10 |
| Stat2 | signal transducer and activator of transcription 2 | -1.81 | -1.66 | 1.72 | 1.83 |
| Sfrp2 | secreted frizzled-related protein 2 | -1.78 | -1.06 | 1.09 | -1.19 |
| Rgs9 | regulator of G-protein signaling 9 | 1.89 | 1.23 | -1.13 | -1.28 |
| 10338690 | #N/A | 1.94 | 1.22 | -1.07 | -1.30 |
| Nptx2 | neuronal pentraxin 2 | 1.97 | 1.25 | -1.51 | -1.50 |
| Prune2 | prune homolog 2 (Drosophila) | 2.04 | 1.44 | -1.51 | 1.56 |
| Tpsb2 | tryptase beta 2 | 2.04 | 1.28 | -1.34 | -1.15 |
| Mcpt4 | mast cell protease 4 | 2.09 | 1.26 | -1.40 | 1.95 |
| Pcdhb2 | protocadherin beta 2 | 2.14 | 1.49 | -2.04 | -1.23 |
| Tnc | tenascin C | 2.17 | 1.79 | -2.17 | -2.06 |
| Ppyr1 | pancreatic polypeptide receptor 1 | 2.20 | 1.61 | -2.15 | -2.59 |
| Gm11428 | predicted gene 11428 | 2.30 | -1.23 | 1.24 | 1.39 |
| D11Bwg0517e | DNA segment, Chr 11, Brigham | 2.32 | 1.71 | -2.21 | -2.39 |
| Slc6a17 | solute carrier family 6 (neurotransmitter transporter), member 17 | 2.41 | 1.52 | -1.99 | -1.50 |
| Art3 | ADP-ribosyltransferase 3 | 2.44 | 2.14 | -2.31 | -1.73 |
| Igfbp2 | insulin-like growth factor binding protein 2 | 2.68 | 1.72 | -2.99 | -2.25 |
| Galnt13 | UDP-N-acetyl-alpha-D-galactosamine:polypeptide N-acetylgalactosaminyltransferase 13 | 2.87 | 2.12 | -2.54 | -3.11 |
| Ces1d | carboxylesterase 1D | 3.01 | -2.16 | -1.13 | 1.88 |
| Cpa3 | carboxypeptidase A3, mast cell | 3.28 | 1.53 | -1.81 | 1.92 |

**Table S6 Molecular and cellular functions associated with the ageing mouse myometrial transcriptome. (28-30 compared with 10-12 weeks).**

Functional enrichment in IPA is based on the remaining 50 transcript cluster IDs following removal of replicate transcript cluster IDs for a given transcript. The transcripts were enriched in the following molecular and cellular function: protein synthesis (10 transcripts, p-value: 0.0003-0.04), cell death (10 transcripts sets, p-value 0.0005-0.05), cell morphology (9 transcripts, p-value 0.0002-0.03), DNA replication, recombination and repair (p-value: 0.001-0.04), and gene expression (7 transcripts, 0.003-0.04). The p-value of the overlap is determined by Fisher’s exact test and is expressed as a range since subcategories exist within the specified function.

| **Protein synthesis (10 transcripts, p-value 0.0003-0.04 )** | | | |
| --- | --- | --- | --- |
| **Affymetrix ID** | **Symbol** | **Entrez Gene Name** | **Type(s)** |
| 10541307 | USP18 | ubiquitin specific peptidase 18 | peptidase |
| 10391207 | DHX58 | DEXH (Asp-Glu-X-His) box polypeptide 58 | enzyme |
| 10399710 | RSAD2 | radical S-adenosyl methionine domain containing 2 | enzyme |
| 10346191 | STAT1 | signal transducer and activator of transcription 1, 91kDa | transcription regulator |
| 10599001 | AGTR2 | angiotensin II receptor, type 2 | G-protein coupled receptor |
| 10420247 | Mcpt4 | mast cell protease 4 | peptidase |
| 10418898 | PPYR1 | pancreatic polypeptide receptor 1 | G-protein coupled receptor |
| 10347277 | IGFBP2 | insulin-like growth factor binding protein 2, 36kDa | other |
| 10580635 | CES1 | carboxylesterase 1D | enzyme |
| 10497451 | CPA3 | carboxypeptidase A3 (mast cell) | peptidase |
| **Cell death (10 transcripts, p-value 0.0005-0.05 )** | | | |
| **Affymetrix ID** | **Symbol** | **Entrez Gene Name** | **Type(s)** |
| 10350173 | TNNT2 | troponin T type 2 (cardiac) | other |
| 10437224 | MX1 | myxovirus (influenza virus) resistance 1 | enzyme |
| 10525158 | Oas1b | 2'-5' oligoadenylate synthetase 1B | enzyme |
| 10378068 | XAF1 | XIAP associated factor 1 | other |
| 10562761 | CLEC11A | C-type lectin domain family 11, member A | growth factor |
| 10346191 | STAT1 | signal transducer and activator of transcription 1, 91kDa | transcription regulator |
| 10599001 | AGTR2 | angiotensin II receptor, type 2 | G-protein coupled receptor |
| 10492798 | SFRP2 | secreted frizzled-related protein 2 | transmembrane receptor |
| 10442786 | TPSAB1/TPSB2 | tryptase alpha/beta 1 | peptidase |
| 10347277 | IGFBP2 | insulin-like growth factor binding protein 2, 36kDa | other |
| **Cell morphology (9 transcripts, p-value 0.002-0.03)** | | | |
| **Affymetrix ID** | **Symbol** | **Entrez Gene Name** | **Type(s)** |
| 10455961 | Iigp1/Iigp1b | Interferon inducible GTPase1 | enzyme |
| 10562761 | CLEC11A | C-type lectin domain family 11, member A | growth factor |
| 10346191 | STAT1 | signal transducer and activator of transcription 1, 91kDa | transcription regulator |
| 10599001 | AGTR2 | angiotensin II receptor, type 2 | G-protein coupled receptor |
| 10420247 | Mcpt4 | mast cell protease 4 | peptidase |
| 10513739 | TNC | tenascin C | other |
| 10347277 | IGFBP2 | insulin-like growth factor binding protein 2, 36kDa | other |
| 10580635 | CES1 | carboxylesterase 1D | enzyme |
| 10497451 | CPA3 | carboxypeptidase A3 (mast cell) | peptidase |
| **DNA replication (7 transcripts, p-value: 0.001-0.04)** | | | |
| **Affymetrix ID** | **Symbol** | **Entrez Gene Name** | **Type(s)** |
| 10599001 | AGTR2 | angiotensin II receptor, type 2 | G-protein coupled receptor |
| 10347277 | IGFBP2 | insulin-like growth factor binding protein 2, 36kDa | other |
| 10455961 | Iigp1/Iigp1b | N/A | enzyme |
| 10525158 | Oas1b | 2'-5' oligoadenylate synthetase 1B | enzyme |
| 10346191 | STAT1 | signal transducer and activator of transcription 1, 91kDa | transcription regulator |
| 10513739 | TNC | tenascin C | other |
| 10442786 | TPSAB1/TPSB2 | tryptase alpha/beta 1 | peptidase |
| **Gene Expression (7 transcripts, p-value: 0.003-0.04)** | | | |
| **Affymetrix ID** | **Symbol** | **Entrez Gene Name** | **Type(s)** |
| 10347277 | IGFBP2 | insulin-like growth factor binding protein 2, 36kDa | other |
| 10569102 | IRF7 | interferon regulatory factor 7 | transcription regulator |
| 10437224 | MX1 | myxovirus (influenza virus) resistance 1 | enzyme |
| 10533246 | OAS1 | 2'-5'-oligoadenylate synthetase 1, 40/46kDa | enzyme |
| 10346191 | STAT1 | signal transducer and activator of transcription 1, 91kDa | transcription regulator |
| 10367224 | STAT2 | signal transducer and activator of transcription 2, 113kDa | transcription regulator |
| 10541307 | USP18 | ubiquitin specific peptidase 18 | peptidase |

**Table S7 Molecular and cellular functions associated with the ageing myometrial transcriptome (38-40 compared with 10-12 weeks).**

Functional enrichment in IPA is based on the remaining 42 transcript cluster IDs following removal of replicated transcript IDs for a given transcript. The transcripts were enriched in the following molecular and cellular functions small molecule biochemistry (15 transcripts, p=0.0005-0.05), Cellular movement (10 transcripts, p-value 0.0005-0.05), Cell Morphology (9 transcripts, p-value=0.0002-0.03), Molecular transport (8 transcripts, p-value=0.0007-0.05), Cell signaling (7 transcripts, p-value=0.0035-0.02). The p-value of the overlap is determined by Fisher’s exact test and is expressed as a range since subcategories exist within the specified function.

| **Small molecule biochemistry (15 transcripts, p-value 0.0005-0.05)** | | | | | |
| --- | --- | --- | --- | --- | --- |
| **Affymetrix ID** | **Symbol** | **Entrez Gene Name** | | | **Type(s)** |
| 10599001 | AGTR2 | angiotensin II receptor, type 2 | | | G-protein coupled receptor |
| 10422164 | EDNRB | endothelin receptor type B | | | G-protein coupled receptor |
| 10531415 | CXCL10 | chemokine (C-X-C motif) ligand 10 | | | cytokine |
| 10566026 | FOLR2 | folate receptor 2 (fetal) | | | transporter |
| 10537062 | MEST | mesoderm specific transcript homolog (mouse) | | | peptidase |
| 10455961 | Iigp1/Iigp1b | -- | | | enzyme |
| 10562761 | CLEC11A | C-type lectin domain family 11, member A | | | growth factor |
| 10441233 | MX1 | myxovirus (influenza virus) resistance 1, interferon-inducible protein p78 (mouse) | | | enzyme |
| 10381809 | ITGB3 | integrin, beta 3 (platelet glycoprotein IIIa, antigen CD61) | | | transmembrane receptor |
| 10513739 | TNC (includes EG:116640) | tenascin C | | | other |
| 10598493 | PCSK1N | proprotein convertase subtilisin/kexin type 1 inhibitor | | | other |
| 10435704 | CD80 (includes EG:12519) | CD80 molecule | | | transmembrane receptor |
| 10360415 | GREM2 | gremlin 2 | | | other |
| 10553967 | PCSK6 | proprotein convertase subtilisin/kexin type 6 | | | peptidase |
| 10355813 | PTPRN | protein tyrosine phosphatase, receptor type, N | | | phosphatase |
| **Cellular movement (10 transcripts, p-value 0.0005-0.05)** | | | | | |
| **Affymetrix ID** | **Symbol** | **Entrez Gene Name** | | **Type(s)** | |
| 10599001 | AGTR2 | angiotensin II receptor, type 2 | | G-protein coupled receptor | |
| 10422164 | EDNRB | endothelin receptor type B | | G-protein coupled receptor | |
| 10531415 | CXCL10 | chemokine (C-X-C motif) ligand 10 | | cytokine | |
| 10562761 | CLEC11A | C-type lectin domain family 11, member A | | growth factor | |
| 10441233 | MX1 | myxovirus (influenza virus) resistance 1, interferon-inducible protein p78 (mouse) | | enzyme | |
| 10355984 | SERPINE2 | serpin peptidase inhibitor, clade E (nexin, plasminogen activator inhibitor type 1), member 2 | | other | |
| 10381809 | ITGB3 | integrin, beta 3 (platelet glycoprotein IIIa, antigen CD61) | | transmembrane receptor | |
| 10513739 | TNC (includes EG:116640) | tenascin C | | other | |
| 10435704 | CD80 (includes EG:12519) | CD80 molecule | | transmembrane receptor | |
| 10553967 | PCSK6 | proprotein convertase subtilisin/kexin type 6 | | peptidase | |
| **Cell Morphology (9 transcripts, p-value=0.0002-0.03)** | | | | | |
| **Affymetrix ID** | **Symbol** | | **Entrez Gene Name** | **Type(s)** | |
| 10599001 | AGTR2 | | angiotensin II receptor, type 2 | G-protein coupled receptor | |
| 10422164 | EDNRB | | endothelin receptor type B | G-protein coupled receptor | |
| 10531415 | CXCL10 | | chemokine (C-X-C motif) ligand 10 | cytokine | |
| 10537062 | MEST | | mesoderm specific transcript homolog (mouse) | peptidase | |
| 10562761 | CLEC11A | | C-type lectin domain family 11, member A | growth factor | |
| 10355984 | SERPINE2 | | serpin peptidase inhibitor, clade E (nexin, plasminogen activator inhibitor type 1), member 2 | other | |
| 10381809 | ITGB3 | | integrin, beta 3 (platelet glycoprotein IIIa, antigen CD61) | transmembrane receptor | |
| 10513739 | TNC (includes EG:116640) | | tenascin C | other | |
| 10435704 | CD80 (includes EG:12519) | | CD80 molecule | transmembrane receptor | |
| **Molecular transport (8 transcripts, p-value=0.0007-0.05)** | | | | | |
| **Affymetrix ID** | **Symbol** | | **Entrez Gene Name** | **Type(s)** | |
| 10599001 | AGTR2 | | angiotensin II receptor, type 2 | G-protein coupled receptor | |
| 10422164 | EDNRB | | endothelin receptor type B | G-protein coupled receptor | |
| 10531415 | CXCL10 | | chemokine (C-X-C motif) ligand 10 | cytokine | |
| 10566026 | FOLR2 | | folate receptor 2 (fetal) | transporter | |
| 10562761 | CLEC11A | | C-type lectin domain family 11, member A | growth factor | |
| 10381809 | ITGB3 | | integrin, beta 3 (platelet glycoprotein IIIa, antigen CD61) | transmembrane receptor | |
| 10513739 | TNC (includes EG:116640) | | tenascin C | other | |
| 10355813 | PTPRN | | protein tyrosine phosphatase, receptor type, N | phosphatase | |
| Cell Signaling (7 transcripts, p-value=0.0035-0.02) | | | | | |
| **Affymetrix ID** | **Symbol** | | **Entrez Gene Name** | **Type(s)** | |
| 10569102 | IRF7 | | interferon regulatory factor 7 | transcription regulator | |
| 10599001 | AGTR2 | | angiotensin II receptor, type 2 | G-protein coupled receptor | |
| 10422164 | EDNRB | | endothelin receptor type B | G-protein coupled receptor | |
| 10531415 | CXCL10 | | chemokine (C-X-C motif) ligand 10 | cytokine | |
| 10455961 | Iigp1/Iigp1b | | -- | enzyme | |
| 10562761 | CLEC11A | | C-type lectin domain family 11, member A | growth factor | |
| 10571815 | GPM6A | | glycoprotein M6A | ion channel | |
| 10355984 | SERPINE2 | | serpin peptidase inhibitor, clade E (nexin, plasminogen activator inhibitor type 1), member 2 | other | |
| 10381809 | ITGB3 | | integrin, beta 3 (platelet glycoprotein IIIa, antigen CD61) | transmembrane receptor | |
| 10513739 | TNC (includes EG:116640) | | tenascin C | other | |
| 10435704 | CD80 (includes EG:12519) | | CD80 molecule | transmembrane receptor | |
| 10352798 | KCNH1 | | potassium voltage-gated channel, subfamily H (eag-related), member 1 | ion channel | |
| 10355813 | PTPRN | | protein tyrosine phosphatase, receptor type, N | phosphatase | |

**Table S8 Molecular and cellular functions associated with ovarian suppression by early ovariectomy.**

Functional enrichment in IPA is based on the remaining 121 transcript cluster IDs following removal of replicated transcript cluster IDs for a given transcript. The transcripts were enriched in the following molecular and cellular functions: cellular growth and development (47 transcripts, p-value: 7.51 x 10^-7^-- 0.0067), cell to cell signaling and interaction (42 transcripts, p-value 3.03 x 10^-9^-0.0051), cellular development (39 transcripts, p-value 7.51 x 10^-7^- 0.0067 ), cellular movement (34 transcripts, p-value:1.28 x 10^-7^- 0.007 ), and protein synthesis (17 transcripts, p value 9.67 x 10^-11^ – 0.0051). Cellular growth and development (47 transcripts, p-value: 7.51 x 10^-7^ - 0.0067). The p-value of the overlap is determined by Fisher’s exact test and is expressed as a range since subcategories exist within the specified function.

| Cellular growth and development (47 transcripts, p-value: 7.51 x 10-7-- 0.0067) | | | |
| --- | --- | --- | --- |
| **Affymetrix ID** | **Symbol** | **Entrez Gene Name** | **Type(s)** |
| 10403069 | IGHM | immunoglobulin heavy constant mu | transmembrane receptor |
| 10562761 | CLEC11A | C-type lectin domain family 11, member A | growth factor |
| 10591781 | ANLN | anillin, actin binding protein | other |
| 10389207 | CCL5 | chemokine (C-C motif) ligand 5 | cytokine |
| 10454709 | KIF20A | kinesin family member 20A | transporter |
| 10487823 | SIGLEC1 | sialic acid binding Ig-like lectin 1, sialoadhesin | other |
| 10351509 | FCGR3A | Fc fragment of IgG, low affinity IIIa, receptor (CD16a) | transmembrane receptor |
| 10379636 | SLFN12 | schlafen family member 12 | enzyme |
| 10512470 | CD72 | CD72 molecule | transmembrane receptor |
| 10412126 | IL31RA | interleukin 31 receptor A | transmembrane receptor |
| 10462618 | IFIT3 | interferon-induced protein with tetratricopeptide repeats 3 | other |
| 10541307 | USP18 | ubiquitin specific peptidase 18 | peptidase |
| 10531415 | CXCL10 | chemokine (C-X-C motif) ligand 10 | cytokine |
| 10599001 | AGTR2 | angiotensin II receptor, type 2 | G-protein coupled receptor |
| 10588479 | TLR9 | toll-like receptor 9 | transmembrane receptor |
| 10406928 | CD180 | CD180 molecule | other |
| 10376324 | Gm5431 | predicted gene 5431 | other |
| 10585276 | POU2AF1 | POU class 2 associating factor 1 | transcription regulator |
| 10416181 | STC1 | stanniocalcin 1 | kinase |
| 10598013 | CCR5 | chemokine (C-C motif) receptor 5 (gene/pseudogene) | G-protein coupled receptor |
| 10404606 | LY86 | lymphocyte antigen 86 | other |
| 10430344 | IL2RB | interleukin 2 receptor, beta | transmembrane receptor |
| 10461605 | Ms4a4b (includes others) | membrane-spanning 4-domains, subfamily A, member 4B | other |
| 10494271 | CTSS | cathepsin S | peptidase |
| 10467578 | PIK3AP1 | phosphoinositide-3-kinase adaptor protein 1 | other |
| 10389231 | CCL3L1/CCL3L3 | chemokine (C-C motif) ligand 3-like 1 | cytokine |
| 10541644 | CD163 | CD163 molecule | transmembrane receptor |
| 10362201 | CTGF | connective tissue growth factor | growth factor |
| 10427035 | NR4A1 | nuclear receptor subfamily 4, group A, member 1 | ligand-dependent nuclear receptor |
| 10416406 | HTR2A | 5-hydroxytryptamine (serotonin) receptor 2A, G protein-coupled | G-protein coupled receptor |
| 10356305 | HTR2B | 5-hydroxytryptamine (serotonin) receptor 2B, G protein-coupled | G-protein coupled receptor |
| 10493114 | NES | nestin | other |
| 10536667 | PTPRZ1 | protein tyrosine phosphatase, receptor-type, Z polypeptide 1 | phosphatase |
| 10381809 | ITGB3 | integrin, beta 3 (platelet glycoprotein IIIa, antigen CD61) | transmembrane receptor |
| 10403743 | INHBA | inhibin, beta A | growth factor |
| 10420366 | GJB6 | gap junction protein, beta 6, 30kDa | transporter |
| 10512377 | CCL21 | chemokine (C-C motif) ligand 21 | cytokine |
| 10409282 | ROR2 | receptor tyrosine kinase-like orphan receptor 2 | kinase |
| 10409579 | CXCL14 | chemokine (C-X-C motif) ligand 14 | cytokine |
| 10409222 | SHC3 | SHC (Src homology 2 domain containing) transforming protein 3 | other |
| 10564482 | SYNM | synemin, intermediate filament protein | other |
| 10534667 | SERPINE1 | serpin peptidase inhibitor, clade E (nexin, plasminogen activator inhibitor type 1), member 1 | other |
| 10513739 | TNC (includes EG:116640) | tenascin C | other |
| 10553967 | PCSK6 | proprotein convertase subtilisin/kexin type 6 | peptidase |
| 10607712 | GRPR | gastrin-releasing peptide receptor | G-protein coupled receptor |
| 10490913 | CA3 | carbonic anhydrase III, muscle specific | enzyme |
| 10347277 | IGFBP2 | insulin-like growth factor binding protein 2, 36kDa | other |
| Cell to cell Signaling and interaction (42 transcripts, p-value 3.03 x 10-9-0.0051) | | | |
| **Affymetrix ID** | **Symbol** | **Entrez Gene Name** | **Type(s)** |
| 10403069 | IGHM | immunoglobulin heavy constant mu | transmembrane receptor |
| 10402864 | IGHA1 | immunoglobulin heavy constant alpha 1 | other |
| 10562761 | CLEC11A | C-type lectin domain family 11, member A | growth factor |
| 10569102 | IRF7 | interferon regulatory factor 7 | transcription regulator |
| 10389207 | CCL5 | chemokine (C-C motif) ligand 5 | cytokine |
| 10487823 | SIGLEC1 | sialic acid binding Ig-like lectin 1, sialoadhesin | other |
| 10351509 | FCGR3A | Fc fragment of IgG, low affinity IIIa, receptor (CD16a) | transmembrane receptor |
| 10603746 | MAOB | monoamine oxidase B | enzyme |
| 10379636 | SLFN12 | schlafen family member 12 | enzyme |
| 10512470 | CD72 | CD72 molecule | transmembrane receptor |
| 10497358 | SIRPB1 | signal-regulatory protein beta 1 | other |
| 10523359 | CXCL13 | chemokine (C-X-C motif) ligand 13 | cytokine |
| 10531415 | CXCL10 | chemokine (C-X-C motif) ligand 10 | cytokine |
| 10599001 | AGTR2 | angiotensin II receptor, type 2 | G-protein coupled receptor |
| 10588479 | TLR9 | toll-like receptor 9 | transmembrane receptor |
| 10406928 | CD180 | CD180 molecule | other |
| 10394054 | CD7 | CD7 molecule | other |
| 10585276 | POU2AF1 | POU class 2 associating factor 1 | transcription regulator |
| 10416181 | STC1 | stanniocalcin 1 | kinase |
| 10392808 | CD300LD | -- | other |
| 10500335 | FCGR1A | Fc fragment of IgG, high affinity Ia, receptor (CD64) | transmembrane receptor |
| 10598013 | CCR5 | chemokine (C-C motif) receptor 5 (gene/pseudogene) | G-protein coupled receptor |
| 10430344 | IL2RB | interleukin 2 receptor, beta | transmembrane receptor |
| 10494271 | CTSS | cathepsin S | peptidase |
| 10389231 | CCL3L1/CCL3L3 | chemokine (C-C motif) ligand 3-like 1 | cytokine |
| 10541644 | CD163 | CD163 molecule | transmembrane receptor |
| 10362201 | CTGF | connective tissue growth factor | growth factor |
| 10427035 | NR4A1 | nuclear receptor subfamily 4, group A, member 1 | ligand-dependent nuclear receptor |
| 10416406 | HTR2A | 5-hydroxytryptamine (serotonin) receptor 2A, G protein-coupled | G-protein coupled receptor |
| 10595324 | HTR1B | 5-hydroxytryptamine (serotonin) receptor 1B, G protein-coupled | G-protein coupled receptor |
| 10381809 | ITGB3 | integrin, beta 3 (platelet glycoprotein IIIa, antigen CD61) | transmembrane receptor |
| 10599927 | AFF2 | AF4/FMR2 family, member 2 | other |
| 10403743 | INHBA | inhibin, beta A | growth factor |
| 10420366 | GJB6 | gap junction protein, beta 6, 30kDa | transporter |
| 10512377 | CCL21 | chemokine (C-C motif) ligand 21 | cytokine |
| 10433887 | PKP2 (includes EG:287925) | plakophilin 2 | other |
| 10409579 | CXCL14 | chemokine (C-X-C motif) ligand 14 | cytokine |
| 10590031 | ITGA9 | integrin, alpha 9 | other |
| 10534667 | SERPINE1 | serpin peptidase inhibitor, clade E (nexin, plasminogen activator inhibitor type 1), member 1 | other |
| 10418898 | PPYR1 (includes EG:19065) | pancreatic polypeptide receptor 1 | G-protein coupled receptor |
| 10513739 | TNC (includes EG:116640) | tenascin C | other |
| 10347277 | IGFBP2 | insulin-like growth factor binding protein 2, 36kDa | other |
| Cellular development ((39 transcripts, p-value 7.51 x 10-7- 0.0067) | | | |
| **Affymetrix ID** | **Symbol** | **Entrez Gene Name** | **Type(s)** |
| 10403069 | IGHM | immunoglobulin heavy constant mu | transmembrane receptor |
| 10562761 | CLEC11A | C-type lectin domain family 11, member A | growth factor |
| 10569102 | IRF7 | interferon regulatory factor 7 | transcription regulator |
| 10389207 | CCL5 | chemokine (C-C motif) ligand 5 | cytokine |
| 10399710 | RSAD2 | radical S-adenosyl methionine domain containing 2 | enzyme |
| 10487823 | SIGLEC1 | sialic acid binding Ig-like lectin 1, sialoadhesin | other |
| 10351509 | FCGR3A | Fc fragment of IgG, low affinity IIIa, receptor (CD16a) | transmembrane receptor |
| 10512470 | CD72 | CD72 molecule | transmembrane receptor |
| 10412126 | IL31RA | interleukin 31 receptor A | transmembrane receptor |
| 10531415 | CXCL10 | chemokine (C-X-C motif) ligand 10 | cytokine |
| 10599001 | AGTR2 | angiotensin II receptor, type 2 | G-protein coupled receptor |
| 10588479 | TLR9 | toll-like receptor 9 | transmembrane receptor |
| 10406928 | CD180 | CD180 molecule | other |
| 10585276 | POU2AF1 | POU class 2 associating factor 1 | transcription regulator |
| 10416181 | STC1 | stanniocalcin 1 | kinase |
| 10500335 | FCGR1A | Fc fragment of IgG, high affinity Ia, receptor (CD64) | transmembrane receptor |
| 10598013 | CCR5 | chemokine (C-C motif) receptor 5 (gene/pseudogene) | G-protein coupled receptor |
| 10404606 | LY86 | lymphocyte antigen 86 | other |
| 10430344 | IL2RB | interleukin 2 receptor, beta | transmembrane receptor |
| 10461605 | Ms4a4b (includes others) | membrane-spanning 4-domains, subfamily A, member 4B | other |
| 10467578 | PIK3AP1 | phosphoinositide-3-kinase adaptor protein 1 | other |
| 10389231 | CCL3L1/CCL3L3 | chemokine (C-C motif) ligand 3-like 1 | cytokine |
| 10541644 | CD163 | CD163 molecule | transmembrane receptor |
| 10502240 | NPNT | nephronectin | other |
| 10362201 | CTGF | connective tissue growth factor | growth factor |
| 10427035 | NR4A1 | nuclear receptor subfamily 4, group A, member 1 | ligand-dependent nuclear receptor |
| 10356305 | HTR2B | 5-hydroxytryptamine (serotonin) receptor 2B, G protein-coupled | G-protein coupled receptor |
| 10493114 | NES | nestin | other |
| 10536667 | PTPRZ1 | protein tyrosine phosphatase, receptor-type, Z polypeptide 1 | phosphatase |
| 10482500 | RND3 | Rho family GTPase 3 | enzyme |
| 10381809 | ITGB3 | integrin, beta 3 (platelet glycoprotein IIIa, antigen CD61) | transmembrane receptor |
| 10403743 | INHBA | inhibin, beta A | growth factor |
| 10512377 | CCL21 | chemokine (C-C motif) ligand 21 | cytokine |
| 10409282 | ROR2 | receptor tyrosine kinase-like orphan receptor 2 | kinase |
| 10409222 | SHC3 | SHC (Src homology 2 domain containing) transforming protein 3 | other |
| 10534667 | SERPINE1 | serpin peptidase inhibitor, clade E (nexin, plasminogen activator inhibitor type 1), member 1 | other |
| 10513739 | TNC (includes EG:116640) | tenascin C | other |
| 10363921 | PCDH15 | protocadherin-related 15 | other |
| 10347277 | IGFBP2 | insulin-like growth factor binding protein 2, 36kDa | other |
| Cellular movement (34 transcripts, p-value:1.28 x 10^-7^- 0.007 ) | | | |
| **Affymetrix ID** | **Symbol** | **Entrez Gene Name** | **Type(s)** |
| 10403069 | IGHM | immunoglobulin heavy constant mu | transmembrane receptor |
| 10562761 | CLEC11A | C-type lectin domain family 11, member A | growth factor |
| 10389207 | CCL5 | chemokine (C-C motif) ligand 5 | cytokine |
| 10351509 | FCGR3A | Fc fragment of IgG, low affinity IIIa, receptor (CD16a) | transmembrane receptor |
| 10379636 | SLFN12 | schlafen family member 12 | enzyme |
| 10523359 | CXCL13 | chemokine (C-X-C motif) ligand 13 | cytokine |
| 10531415 | CXCL10 | chemokine (C-X-C motif) ligand 10 | cytokine |
| 10599001 | AGTR2 | angiotensin II receptor, type 2 | G-protein coupled receptor |
| 10588479 | TLR9 | toll-like receptor 9 | transmembrane receptor |
| 10585276 | POU2AF1 | POU class 2 associating factor 1 | transcription regulator |
| 10501063 | CD53 | CD53 molecule | other |
| 10416181 | STC1 | stanniocalcin 1 | kinase |
| 10598013 | CCR5 | chemokine (C-C motif) receptor 5 (gene/pseudogene) | G-protein coupled receptor |
| 10430344 | IL2RB | interleukin 2 receptor, beta | transmembrane receptor |
| 10494271 | CTSS | cathepsin S | peptidase |
| 10389231 | CCL3L1/CCL3L3 | chemokine (C-C motif) ligand 3-like 1 | cytokine |
| 10362201 | CTGF | connective tissue growth factor | growth factor |
| 10356305 | HTR2B | 5-hydroxytryptamine (serotonin) receptor 2B, G protein-coupled | G-protein coupled receptor |
| 10493114 | NES | nestin | other |
| 10536667 | PTPRZ1 | protein tyrosine phosphatase, receptor-type, Z polypeptide 1 | phosphatase |
| 10482500 | RND3 | Rho family GTPase 3 | enzyme |
| 10381809 | ITGB3 | integrin, beta 3 (platelet glycoprotein IIIa, antigen CD61) | transmembrane receptor |
| 10403743 | INHBA | inhibin, beta A | growth factor |
| 10487797 | ADAM33 | ADAM metallopeptidase domain 33 | peptidase |
| 10512377 | CCL21 | chemokine (C-C motif) ligand 21 | cytokine |
| 10409282 | ROR2 | receptor tyrosine kinase-like orphan receptor 2 | kinase |
| 10409579 | CXCL14 | chemokine (C-X-C motif) ligand 14 | cytokine |
| 10590031 | ITGA9 | integrin, alpha 9 | other |
| 10564482 | SYNM | synemin, intermediate filament protein | other |
| 10534667 | SERPINE1 | serpin peptidase inhibitor, clade E (nexin, plasminogen activator inhibitor type 1), member 1 | other |
| 10513739 | TNC (includes EG:116640) | tenascin C | other |
| 10553967 | PCSK6 | proprotein convertase subtilisin/kexin type 6 | peptidase |
| 10490913 | CA3 | carbonic anhydrase III, muscle specific | enzyme |
| 10347277 | IGFBP2 | insulin-like growth factor binding protein 2, 36kDa | other |
| Protein synthesis (17 transcripts, p value 9.67 x 10^-11^ – 0.0051) | | | |
| **Affymetrix ID** | **Symbol** | **Entrez Gene Name** | **Type(s)** |
| 10403069 | IGHM | immunoglobulin heavy constant mu | transmembrane receptor |
| 10531126 | IGJ | immunoglobulin J polypeptide, linker protein for immunoglobulin alpha and mu polypeptides | other |
| 10399710 | RSAD2 | radical S-adenosyl methionine domain containing 2 | enzyme |
| 10487823 | SIGLEC1 | sialic acid binding Ig-like lectin 1, sialoadhesin | other |
| 10512470 | CD72 | CD72 molecule | transmembrane receptor |
| 10412126 | IL31RA | interleukin 31 receptor A | transmembrane receptor |
| 10531415 | CXCL10 | chemokine (C-X-C motif) ligand 10 | cytokine |
| 10588479 | TLR9 | toll-like receptor 9 | transmembrane receptor |
| 10406928 | CD180 | CD180 molecule | other |
| 10585276 | POU2AF1 | POU class 2 associating factor 1 | transcription regulator |
| 10500335 | FCGR1A | Fc fragment of IgG, high affinity Ia, receptor (CD64) | transmembrane receptor |
| 10598013 | CCR5 | chemokine (C-C motif) receptor 5 (gene/pseudogene) | G-protein coupled receptor |
| 10404606 | LY86 | lymphocyte antigen 86 | other |
| 10430344 | IL2RB | interleukin 2 receptor, beta | transmembrane receptor |
| 10494271 | CTSS | cathepsin S | peptidase |
| 10467578 | PIK3AP1 | phosphoinositide-3-kinase adaptor protein 1 | other |
| 10381809 | ITGB3 | integrin, beta 3 (platelet glycoprotein IIIa, antigen CD61) | transmembrane receptor |

**Table S9 Molecular and cellular functions associated with transcripts differentially regulated by long-term progesterone treatment.**

Functional enrichment in IPA is based on the remaining 85 transcript cluster IDs following removal of duplicated probes for a given transcript. The transcripts were enriched in the following molecular and cellular functions cellular growth and proliferation (28 transcripts, p-value: 0.00002-0.05), cell-to-cell signaling and interaction (21 transcripts sets, p-value: 0.00002-0.05), protein synthesis (13 transcripts, p-value: 0.000003-0.03), nucleic acid metabolism (11 transcripts, p-value: 0.00002-0.03), and drug metabolism (4 transcripts, p-value: 0.00002-0.04). The p-value of the overlap is determined by Fisher’s exact test and is expressed as a range since subcategories exist within the specified function.

| **Cellular growth and proliferation (28 transcripts, p-value: 0.00002-0.05)** | | | | | |
| --- | --- | --- | --- | --- | --- |
| **Affymetrix ID** | **Symbol** | | **Entrez Gene Name** | | **Type(s)** |
| 10379636 | SLFN12 | | schlafen family member 12 | | enzyme |
| 10379633 | Slfn1 | | schlafen 1 | | enzyme |
| 10533213 | OAS3 | | 2'-5'-oligoadenylate synthetase 3, 100kDa | | enzyme |
| 10462618 | IFIT3 | | interferon-induced protein with tetratricopeptide repeats 3 | | other |
| 10452316 | C3 | | complement component 3 | | peptidase |
| 10541307 | USP18 | | ubiquitin specific peptidase 18 | | peptidase |
| 10461594 | Ms4a4b (includes others) | | membrane-spanning 4-domains, subfamily A, member 4B | | other |
| 10441233 | MX1 | | myxovirus (influenza virus) resistance 1, interferon-inducible protein p78 (mouse) | | enzyme |
| 10376324 | Gm5431 | | predicted gene 5431 | | other |
| 10566026 | FOLR2 | | folate receptor 2 (fetal) | | transporter |
| 10385500 | IRGM | | immunity-related GTPase family, M | | other |
| 10487823 | SIGLEC1 | | sialic acid binding Ig-like lectin 1, sialoadhesin | | other |
| 10351509 | FCGR3A | | Fc fragment of IgG, low affinity IIIa, receptor (CD16a) | | transmembrane receptor |
| 10458560 | FGF1 | | fibroblast growth factor 1 (acidic) | | growth factor |
| 10443195 | HMGA1 | | -- | | transcription regulator |
| 10473356 | UBE2L6 | | ubiquitin-conjugating enzyme E2L 6 | | enzyme |
| 10429564 | Ly6a (includes others) | | lymphocyte antigen 6 complex, locus A | | other |
| 10588479 | TLR9 | | toll-like receptor 9 | | transmembrane receptor |
| 10444244 | TAP1 | | transporter 1, ATP-binding cassette, sub-family B (MDR/TAP) | | transporter |
| 10523012 | DCK | | deoxycytidine kinase | | kinase |
| 10483110 | IFIH1 | | interferon induced with helicase C domain 1 | | enzyme |
| 10346191 | STAT1 | | signal transducer and activator of transcription 1, 91kDa | | transcription regulator |
| 10347277 | IGFBP2 | | insulin-like growth factor binding protein 2, 36kDa | | other |
| 10553967 | PCSK6 | | proprotein convertase subtilisin/kexin type 6 | | peptidase |
| 10352798 | KCNH1 | | potassium voltage-gated channel, subfamily H (eag-related), member 1 | | ion channel |
| 10420366 | GJB6 | | gap junction protein, beta 6, 30kDa | | transporter |
| 10355813 | PTPRN | | protein tyrosine phosphatase, receptor type, N | | phosphatase |
| 10607712 | GRPR | | gastrin-releasing peptide receptor | | G-protein coupled receptor |
| **Cell to cell signaling and interaction ((21 transcripts sets, p-value: 0.00002-0.05)** | | | | | |
| **Affymetrix ID** | **Symbol** | **Entrez Gene Name** | | | **Type(s)** |
| 10569102 | IRF7 | interferon regulatory factor 7 | | | transcription regulator |
| 10452316 | C3 | complement component 3 | | | peptidase |
| 10542470 | MGST1 | microsomal glutathione S-transferase 1 | | | enzyme |
| 10500335 | FCGR1A | Fc fragment of IgG, high affinity Ia, receptor (CD64) | | | transmembrane receptor |
| 10455961 | Iigp1/Iigp1b | -- | | | enzyme |
| 10385500 | IRGM | immunity-related GTPase family, M | | | other |
| 10487823 | SIGLEC1 | sialic acid binding Ig-like lectin 1, sialoadhesin | | | other |
| 10351509 | FCGR3A | Fc fragment of IgG, low affinity IIIa, receptor (CD16a) | | | transmembrane receptor |
| 10458560 | FGF1 | fibroblast growth factor 1 (acidic) | | | growth factor |
| 10473356 | UBE2L6 | ubiquitin-conjugating enzyme E2L 6 | | | enzyme |
| 10429564 | Ly6a (includes others) | lymphocyte antigen 6 complex, locus A | | | other |
| 10588479 | TLR9 | toll-like receptor 9 | | | transmembrane receptor |
| 10444244 | TAP1 | transporter 1, ATP-binding cassette, sub-family B (MDR/TAP) | | | transporter |
| 10483110 | IFIH1 | interferon induced with helicase C domain 1 | | | enzyme |
| 10346191 | STAT1 | signal transducer and activator of transcription 1, 91kDa | | | transcription regulator |
| 10489569 | PLTP | phospholipid transfer protein | | | other |
| 10347277 | IGFBP2 | insulin-like growth factor binding protein 2, 36kDa | | | other |
| 10418898 | PPYR1 (includes EG:19065) | pancreatic polypeptide receptor 1 | | | G-protein coupled receptor |
| 10352798 | KCNH1 | potassium voltage-gated channel, subfamily H (eag-related), member 1 | | | ion channel |
| 10420366 | GJB6 | gap junction protein, beta 6, 30kDa | | | transporter |
| 10355813 | PTPRN | protein tyrosine phosphatase, receptor type, N | | | phosphatase |
| **Protein synthesis (13 transcripts, p-value: 0.000003-0.03)** | | | | | |
| **Affymetrix ID** | **Symbol** | | | **Entrez Gene Name** | **Type(s)** |
| 10452316 | C3 | | | complement component 3 | peptidase |
| 10399710 | RSAD2 | | | radical S-adenosyl methionine domain containing 2 | enzyme |
| 10391207 | DHX58 | | | DEXH (Asp-Glu-X-His) box polypeptide 58 | enzyme |
| 10385500 | IRGM | | | immunity-related GTPase family, M | other |
| 10376326 | Igtp | | | interferon gamma induced GTPase | enzyme |
| 10443195 | HMGA1 | | | -- | transcription regulator |
| 10444244 | TAP1 | | | transporter 1, ATP-binding cassette, sub-family B (MDR/TAP) | transporter |
| 10346191 | STAT1 | | | signal transducer and activator of transcription 1, 91kDa | transcription regulator |
| 10489569 | PLTP | | | phospholipid transfer protein | other |
| 10607705 | S100G | | | S100 calcium binding protein G | other |
| 10347277 | IGFBP2 | | | insulin-like growth factor binding protein 2, 36kDa | other |
| 10553967 | PCSK6 | | | proprotein convertase subtilisin/kexin type 6 | peptidase |
| 10418898 | PPYR1 (includes EG:19065) | | | pancreatic polypeptide receptor 1 | G-protein coupled receptor |

| **Nucleic acid metabolism (11 transcripts, p-value: 0.00002-0.03)** | | | | |
| --- | --- | --- | --- | --- |
| **Affymetrix ID** | | **Symbol** | **Entrez Gene Name** | **Type(s)** |
| 10533256 | | OAS1 | 2'-5'-oligoadenylate synthetase 1, 40/46kDa | enzyme |
| 10533213 | | OAS3 | 2'-5'-oligoadenylate synthetase 3, 100kDa | enzyme |
| 10533198 | | OAS2 | 2'-5'-oligoadenylate synthetase 2, 69/71kDa | enzyme |
| 10455961 | | Iigp1/Iigp1b | -- | enzyme |
| 10385533 | | Tgtp1 | -- | enzyme |
| 10376326 | | Igtp | interferon gamma induced GTPase | enzyme |
| 10395039 | | CMPK2 | cytidine monophosphate (UMP-CMP) kinase 2, mitochondrial | kinase |
| 10444244 | | TAP1 | transporter 1, ATP-binding cassette, sub-family B (MDR/TAP) | transporter |
| 10523012 | | DCK | deoxycytidine kinase | kinase |
| 10418898 | | PPYR1 (includes EG:19065) | pancreatic polypeptide receptor 1 | G-protein coupled receptor |
| 10607712 | | GRPR | gastrin-releasing peptide receptor | G-protein coupled receptor |
| **Drug metabolism (4 transcripts, p-value: 0.00002-0.04)** | | | | |
| Affymetrix | Symbol | | Entrez Gene Name | Type(s) |
| 10566026 | FOLR2 | | folate receptor 2 (fetal) | transporter |
| 10395039 | CMPK2 | | cytidine monophosphate (UMP-CMP) kinase 2, mitochondrial | kinase |
| 10523012 | DCK | | deoxycytidine kinase | kinase |
| 10355813 | PTPRN | | protein tyrosine phosphatase, receptor type, N | phosphatase |

**Table S10 Canonical pathways associated with the ageing myometrial transcriptome (28-30 weeks compared with 10-12 weeks).**

In total, 57 canonical pathways were identified (50 signaling pathways and 7 metabolic pathways). However, only 9 canonical pathways were significant (1 metabolic and 8 signaling pathways. The ratio for a given pathway is the proportion of associated transcripts compared with the total number of transcripts involved in that pathway. The P-value refers to the statistical significance of the overlap calculated by Fisher’s Exact Test with multiple testing correction.

| Ingenuity Canonical Pathways | P-value | Ratio | Associated transcripts |
| --- | --- | --- | --- |
| Interferon Signaling | 3.71535E-08 | 0.139 | IFIT3, OAS1, MX1, STAT2, STAT1 |
| Activation of IRF by Cytosolic Pattern Recognition Receptors | 2.95121E-05 | 0.0556 | DHX58, IRF7, STAT2, STAT1 |
| Role of JAK1, JAK2 and TYK2 in Interferon Signaling | 0.002 | 0.0741 | STAT2, STAT1 |
| Role of Pattern Recognition Receptors in Recognition of Bacteria and Viruses | 0.002 | 0.0283 | OAS1 , IRF7, Oas1b |
| Role of RIG1-like Receptors in Antiviral Innate Immunity | 0.007 | 0.0408 | DHX58, IRF7 |
| JAK/Stat Signaling | 0.015 | 0.0286 | STAT2, STAT1 |
| FLT3 Signaling in Hematopoietic Progenitor Cells | 0.018 | 0.027 | STAT2, STAT1 |
| Lysine Biosynthesis | 0.028 | 0.0156 | VNN1 |
| Renin-Angiotensin Signaling | 0.038 | 0.0159 | STAT1, AGTR2 |

**Table S11 Canonical pathways associated with the ageing myometrial transcriptome (38-40 weeks compared with 10-12 weeks).**

In total, 73 canonical pathways were identified (7 signaling pathways and 2 metabolic pathways). However, only 7 canonical pathways were significant and all were signaling pathways.

| Ingenuity Canonical Pathways | P-value | Ratio | Associated transcripts |
| --- | --- | --- | --- |
| Interferon Signaling | 0.003 | 0.0556 | IFIT3, MX1 |
| Role of RIG1-like Receptors in Antiviral Innate Immunity | 0.003 | 0.0408 | DHX58, IRF7 |
| Activation of IRF by Cytosolic Pattern Recognition Receptors | 0.008 | 0.0278 | DHX58, IRF7 |
| Communication between Innate and Adaptive Immune Cells | 0.013 | 0.0182 | CXCL10, CD80 (includes EG:12519) |
| Pathogenesis of Multiple Sclerosis | 0.021 | 0.1110 | CXCL10 |
| Role of Lipids/Lipid Rafts in the Pathogenesis of Influenza | 0.021 | 0.0357 | RSAD2 |
| Type I Diabetes Mellitus Signaling | 0.03 | 0.0165 | CD80 (includes EG:12519), PTPRN |

**Table S12 Canonical pathways associated with ovarian suppression by early ovariectomy.**

In total, 109 canonical pathways were identified (98 Signaling pathways and 17 metabolic pathways). Of these canonical pathways, 17 were significant and all were Signaling pathways.

| Ingenuity Canonical Pathways | P-value | Ratio | Molecules |
| --- | --- | --- | --- |
| Serotonin Receptor Signaling | 2.51189E-06 | 0.109 | MAOB, HTR2B, HTR1B, HTR1D, HTR2A |
| Pathogenesis of Multiple Sclerosis | 2.39883E-05 | 0.333 | CXCL10, CCR5, CCL5 |
| Communication between Innate and Adaptive Immune Cells | 0.0004 | 0.0455 | CXCL10, CCL3L1/CCL3L3, IGHA1, CCL5, TLR9 |
| Role of Pattern Recognition Receptors in Recognition of Bacteria and Viruses | 0.0005 | 0.0472 | OAS1, IRF7, OAS2, CCL5, TLR9 |
| Hepatic Fibrosis / Hepatic Stellate Cell Activation | 0.003 | 0.034 | CCR5, CTGF, CCL21, MYL4, CCL5 |
| Role of Hypercytokinemia/hyperchemokinemia in the Pathogenesis of Influenza | 0.003 | 0.0682 | CXCL10, CCR5, CCL5 |
| G-Protein Coupled Receptor Signaling | 0.007 | 0.0171 | CCR5, HTR2B, HTR1B, GRPR, HTR1D, BAI3, PPYR1, AGTR2, HTR2A |
| Activation of IRF by Cytosolic Pattern Recognition Receptors | 0.009 | 0.0417 | DHX58, IRF7, ZBP1 |
| IL-17A Signaling in Gastric Cells | 0.012 | 0.08 | CXCL10, CCL5 |
| Systemic Lupus Erythematosus Signaling | 0.017 | 0.0202 | CD72, IGHM, FCGR1A, FCGR3A, TLR9 |
| Altered T Cell and B Cell Signaling in Rheumatoid Arthritis | 0.020 | 0.0326 | CXCL13, CCL21, TLR9 |
| Interferon Signaling | 0.022 | 0.0556 | IFIT3, OAS1 |
| Fcγ Receptor-mediated Phagocytosis in Macrophages and Monocytes | 0.025 | 0.0294 | PLD4, FCGR1A, FCGR3A |
| Role of RIG1-like Receptors in Antiviral Innate Immunity | 0.035 | 0.0408 | DHX58, IRF7 |
| Renin-Angiotensin Signaling | 0.037 | 0.0238 | SHC3, CCL5, AGTR2 |
| Hematopoiesis from Pluripotent Stem Cells | 0.048 | 0.0312 | IGHM, IGHA1 |
| Primary Immunodeficiency Signaling | 0.049 | 0.0317 | IGHM, IGHA1 |

**Table S13 Canonical pathways associated with ovarian suppression by long-term progesterone treatment.**

In total, 98 canonical pathways were identified (6 Signaling pathways and 92 metabolic pathways). However, only 13 (13.3%) canonical pathways were significant and all were Signaling pathways.

| Ingenuity Canonical Pathways | P-value | Ratio | Molecules |
| --- | --- | --- | --- |
| Activation of IRF by Cytosolic Pattern Recognition Receptors | 3.54813E-09 | 0.0972 | DHX58, IFIH1, IRF7, ZBP1, STAT2, STAT1, IFIT2 |
| Interferon Signaling | 4.2658E-09 | 0.167 | IFIT3, OAS1, MX1, STAT2, STAT1, TAP1 |
| Role of Pattern Recognition Receptors in Recognition of Bacteria and Viruses | 1.23027E-08 | 0.0755 | IFIH1,OAS1, IRF7, C3, OAS2, Oas1b, OAS3,TLR9 |
| Role of RIG1-like Receptors in Antiviral Innate Immunity | 0.0004 | 0.0612 | DHX58, IFIH1, IRF7 |
| Dendritic Cell Maturation | 0.0007 | 0.0242 | STAT2, STAT1, FCGR1A, FCGR3A, TLR9 |
| Role of JAK1, JAK2 and TYK2 in Interferon Signaling | 0.005 | 0.0741 | STAT2, STAT1 |
| Role of PKR in Interferon Induction and Antiviral Response | 0.015 | 0.0435 | STAT1, FCGR1A |
| iNOS Signaling | 0.017 | 0.0377 | HMGA1, STAT1 |
| Hepatic Fibrosis / Hepatic Stellate Cell Activation | 0.027 | 0.0204 | MYL4, STAT1, FGF1 |
| Role of Lipids/Lipid Rafts in the Pathogenesis of Influenza | 0.038 | 0.0357 | RSAD2 |
| Systemic Lupus Erythematosus Signaling | 0.040 | 0.0121 | FCGR1A, FCGR3A, TLR9 |
| JAK/Stat Signaling | 0.041 | 0.0286 | STAT2, STAT1 |
| FLT3 Signaling in Hematopoietic Progenitor Cells | 0.043 | 0.027 | STAT2, STAT1 |

**Table S14 Predicted upstream regulators associated with the ageing myometrial transcriptome (28-30 compared with 10-12 weeks).**

7 out of 35 (20%) upstream regulators had a z-score and they are listed. 3 out of the 7 predicted upstream regulators were also differentially regulated by age: Irf7, Stat1 and Stat2.  The table is arranged in order of the fold change of the upstream regulator. This value is available if the upstream regulator predicted was present in the list of transcripts differentially regulated by age. A predicted activation stated is made by IPA if the z-score is greater than 2 or less than -2. The p-value of the overlap is determined by Fisher’s exact test. The target transcripts are those found differentially regulated by ageing myometrium (28-30 compared with 10-12 weeks)

| Upstream Regulator | Fold Change | Molecule Type | Predicted Activation State | z-score | P-value of overlap | Target transcripts |
| --- | --- | --- | --- | --- | --- | --- |
| IRF7 | -2.333 | transcription regulator | Inhibited | -2.324 | 6.4E-16 | DHX58, GBP4, IFI44, IFIT1B, IFIT3, MX1, OAS1, RSAD2, RTP4, STAT1 |
| STAT1 | -1.891 | transcription regulator |  | -1.449 | 0.000000177 | Gm11428, IFIT1B, IFIT3, IRF7, RNF213, RSAD2, STAT1, STAT2, USP18 |
| STAT2 | -1.809 | transcription regulator |  | -1.584 | 0.000000121 | IFIT1B, IFIT3, IRF7, MX1, OAS1 |
| TRIM24 |  | transcription regulator | Activated | 2.419 | 5.88E-19 | DDX60, DHX58, GBP4, IFI27L2, IFI44, IFIT1B, IFIT3, Iigp1/Iigp1b, IRF7, OAS1 |
| IRF3 |  | transcription regulator | Inhibited | -2.106 | 1.25E-08 | DHX58, IFI44, IFIT1B, IFIT3, IRF7, MX1, RSAD2, USP18 |
| IRF |  | transcription regulator | Inhibited | -2.102 | 0.000000052 | AGTR2, IFIT3, IRF7,MX1, OAS1, RSAD2, STAT1, STAT2 |
| IRF9 |  | transcription regulator |  | -1.987 | 0.00000347 | IFIT3, IRF7, STAT1, STAT2 |

**Table S15 Predicted upstream regulators associated with the ageing myometrial transcriptome (38-40 compared with 10-12 weeks).**

10 out of 38 (26.3%) upstream regulators had a z-score. Only one predicted upstream regulator was differentially regulated by age, and this was IRF7. A value for the fold-change is available if the upstream regulator predicted was present in the list of transcripts differentially regulated by age. A predicted activation stated is made by IPA if the z-score is greater than 2 or less than -2 (). p-value of the overlap is determined by Fisher’s exact test. The target transcripts are those found differentially regulated by ageing myometrium (38-40 compared with 10-12 weeks).

| Upstream Regulator | Fold Change | Molecule Type | Predicted Activation State | z-score | P-value of overlap | Target molecules in dataset |
| --- | --- | --- | --- | --- | --- | --- |
| IRF7 | -2.083 | transcription regulator |  | -1.529 | 2.69E-14 | CD80 (includes EG:12519), CXCL10, DHX58, IFI44, IFIT1B, IFIT3, Ms4a4b (includes others), MX1, RSAD2, RTP4, USP18 |
| TRIM24 |  | transcription regulator | Activated | 2.392 | 4.23E-16 | CXCL10, DDX60, DHX58, IFI27L2, IFI44, IFIT1B, IFIT3, Iigp1/Iigp1b, IRF7, Ms4a4b (includes others), RTP4, USP18 |
| IRF3 |  | transcription regulator | Inhibited | -2.482 | 5.22E-13 | CXCL10, DHX58, IFI44, IFIT1B, IFIT3, IRF7, Ms4a4b (includes others), MX1, RSAD2, USP18 |
| STAT2 |  | transcription regulator |  | -1.702 | 2.52E-08 | CXCL10, IFIT1B, IFIT3, IRF7, MX1 |
| STAT1 |  | transcription regulator |  | -1.989 | 7.84E-07 | CXCL10, GBP6, IFIT1B, IFIT3, IRF7, RNF213, RSAD2, USP18 |
| IRF1 |  | transcription regulator |  | -1.726 | 6.03E-06 | AGTR2, CXCL10, IFIT3, IRF7, MX1, RSAD2 |
| RELA |  | transcription regulator |  | 0.557 | 4.88E-04 | CD80 (includes EG:12519), CXCL10, IFIT1B, IRF7, MX1, SERPINE2 |
| BRCA1 |  | transcription regulator |  | -0.911 | 1.01E-03 | IFIT3, IRF7, MX1, SERPINE2 |
| STAT4 |  | transcription regulator |  | -1.179 | 7.65E-03 | CXCL10, IFIT1B, Ms4a4b (includes others), MX1 |
| NFkB (complex) |  | complex |  | -0.591 | 3.15E-02 | CD80 (includes EG:12519), CXCL10, EDNRB, IRF7, RSAD2 |

**Table S16 Predicted upstream regulators associated with early ovariectomy.**

21 out of 80 (26.3%) upstream regulators had a z-score. IRF7 was also predicted to be down-regulated with late ovariectomy and was differentially regulated by late ovariectomy (i.e up-regulated by early ovariectomy). It was also down-regulated by ageing to both 28-30 weeks and 38-40 weeks. A value for the fold-change is available if the upstream regulator predicted was present in the list of transcripts differentially regulated by age. A predicted activation stated is made by IPA if the z-score is greater than 2 or less than -2. The P-value of the overlap is determined by Fisher’s exact test. The target transcripts are those found differentially regulated by ageing myometrium (28-30 compared with 10-12 weeks).

| **Upstream Regulator** | **Fold Change** | **Molecule Type** | **Predicted Activation State** | **z-score** | **P-value of overlap** | **Target molecules in dataset** |
| --- | --- | --- | --- | --- | --- | --- |
| IRF7 | -2.201 | transcription regulator | Inhibited | -2.544 | 4.12E-11 | CCL5, CXCL10, DHX58, IFI44, IFIT1B, IFIT3, Ms4a4b (includes others), OAS1, OAS2, RSAD2, RTP4, USP18, ZBP1 |
| TRIM24 |  | transcription regulator | Activated | 2.683 | 5.03E-15 | CXCL10, DDX60, DHX58, IFI27L2, IFI44, IFIT1B, IFIT3, Iigp1/Iigp1b, IRF7, Ms4a4b (includes others), OAS1, RTP4, SERPINE1, Tgtp1, USP18 |
| IRF3 |  | transcription regulator | Inhibited | -2.287 | 2.53E-10 | CCL3L1/CCL3L3, CCL5, CXCL10, DHX58, IFI44, IFIT1B, IFIT3, IRF7, Ms4a4b (includes others), RSAD2, USP18, ZBP1 |
| STAT3 |  | transcription regulator |  | -1.777 | 7.48E-10 | CCL3L1/CCL3L3, CCL5, CCR5, CXCL10, CXCL13, FCGR1A, HTR2A, IFI44, IFIT1B, IFIT3, IL2RB, IRF7, OAS1, OAS2, SERPINE1, SLFN12, SLFN13, TRIM14 |
| STAT1 |  | transcription regulator | Inhibited | -2.408 | 7.74E-10 | CCL3L1/CCL3L3, CCL5, CXCL10, FCGR1A, GBP6, IFIT1B, IFIT3, IRF7, RNF213, RSAD2, SLFN12, SLFN13, Tgtp1, TLR9, USP18 |
| IRF1 |  | transcription regulator | Inhibited | -2.227 | 2.89E-07 | AGTR2, CCL5, CTSS, CXCL10, IFIT3, IRF7, OAS1, OAS2, RSAD2, TLR9 |
| STAT6 |  | transcription regulator |  | 1.92 | 8.45E-07 | CCL3L1/CCL3L3, CCL5, CCR5, CTSS, CXCL10, FCGR1A, FCGR3A, IFIT3, Ms4a4b (includes others), Tgtp1, Trim30a/Trim30d |
| STAT2 |  | transcription regulator |  | -1.662 | 6.42E-06 | CXCL10,IFIT1B,IFIT3,IRF7,OAS1 |
| IRF5 |  | transcription regulator |  | -0.831 | 2.76E-05 | CCL21,CCL5,IFI44,OAS1,RSAD2 |
| IRF2 |  | transcription regulator |  | 1.113 | 8.29E-05 | CTSS,CXCL10,IRF7,OAS1,USP18 |
| STAT4 |  | transcription regulator |  | -0.012 | 2.77E-03 | CCR5,CXCL10,IFIT1B,Ms4a4b (includes others),PKP2 (includes EG:287925),SERPINE1,Trim30a/Trim30d |
| CREBBP |  | transcription regulator |  | 1.423 | 5.85E-03 | CCL5,CCR5,CXCL10,IL2RB,NR4A1,TNC (includes EG:116640) |
| NFkB (complex) |  | complex |  | -0.773 | 6.17E-03 | CCL3L1/CCL3L3,CCL5,CD7,CTGF,CXCL10,FCGR1A,IGFBP2,Iglv1,IRF7,RSAD2,SERPINE1 |
| IRF8 |  | transcription regulator |  | -0.899 | 6.96E-03 | CCL5,CTSS,OAS1,TLR9 |
| NFKB1 |  | transcription regulator |  | -0.987 | 7.94E-03 | CCL3L1/CCL3L3,CCL5,CXCL10,IFIT1B,IGHM,NR4A1 |
| SMARCB1 |  | transcription regulator |  | 0.841 | 1.38E-02 | FCGR1A,IGFBP2,OAS1,RND3,SGCG |
| SMAD7 |  | transcription regulator | Inhibited | -2.139 | 1.54E-02 | CCL5,CTGF,CXCL10,SERPINE1 |
| RELA |  | transcription regulator |  | 0.267 | 1.66E-02 | CCL5,CXCL10,IFIT1B,IGHM,IRF7,NR4A1,OAS2 |
| SMAD3 |  | transcription regulator |  | 1.617 | 1.71E-02 | CCL3L1/CCL3L3,CTGF,CXCL10,SERPINE1,TNC (includes EG:116640) |
| KLF2 |  | transcription regulator |  | -1.41 | 3.00E-02 | CCR5,CTGF,ITGB3,SERPINE1 |
| EGR1 |  | transcription regulator |  | 0.488 | 3.39E-02 | CCL3L1/CCL3L3,MAOB,NR4A1,SERPINE1 |

**Table S17 Predicted upstream regulators associated with ovarian suppression using progesterone implants.**

14 out of 32 (40.6%) predicted upstream regulators had a z-score and they are listed. This transcript was predicted to be down-regulated by age. A value for the fold-change is available if the upstream regulator predicted was present in the list of transcripts differentially regulated by age. A predicted activation stated is made by IPA if the z-score is greater than 2 or less than -2. The P-value of the overlap is determined by Fisher’s exact test. The target transcripts are those found differentially regulated by ageing myometrium (28-30 compared with 10-12 weeks). *z-score not provided by IPA.

| Upstream Regulator | Fold Change | Molecule Type | Predicted Activation State | z-score | P-value of overlap | Target molecules in dataset |
| --- | --- | --- | --- | --- | --- | --- |
| IRF7 | -2.514 | transcription regulator |  | * | 1.59E-28 | DHX58, GBP4, IFI44, IFIH1, IFIT1B, IFIT2, IFIT3, Ms4a4b (includes others), MX1, OAS1, OAS2, OAS3, OASL, RSAD2, RTP4, STAT1, STAT2, TAP1, UBE2L6, USP18, XAF1, ZBP1 |
| STAT1 | -1.735 | transcription regulator | Inhibited | -2.409 | 9.43E-21 | C3, CMPK2, FCGR1A, GBP6, HERC6, IFIT1B, IFIT2, IFIT3, Igtp, IRF7, IRGM, RNF213, RSAD2, Slfn1, SLFN12, SLFN13, STAT1, STAT2, TAP1, Tgtp1, TLR9, USP18 |
| IRF3 |  | transcription regulator | Inhibited | -2.102 | 8.06E-16 | DHX58, FGF1, IFI44, IFIH1, IFIT1B, IFIT2, IFIT3, IRF7, Ms4a4b (includes others), MX1, OAS3, RSAD2, USP18, ZBP1 |
| STAT3 |  | transcription regulator |  | -1.14 | 4.06E-11 | FCGR1A, IFI44, IFIH1, IFIT1B, IFIT3, IRF7, MX1, OAS1, OAS2, OAS3, OASL, Slfn1, SLFN12, SLFN13, STAT1, TAP1, XAF1 |
| IRF1 |  | transcription regulator |  | -1.766 | 4.73E-11 | IFIH1, IFIT2, IFIT3, IRF7, MX1, OAS1, OAS2, RSAD2, STAT1, STAT2, TAP1, TLR9 |
| STAT2 | -1.828 | transcription regulator |  | -1.409 | 1.37E-08 | IFIT1B, IFIT2, IFIT3,IRF7, MX1, OAS1 |
| ISGF3 |  | complex |  |  | 8.62E-08 | IFIH1, IFIT2, IRF7, RSAD2 |
| IRF9 |  | transcription regulator |  | -1.568 | 5.16E-07 | IFIT3, IRF7, STAT1, STAT2, Tgtp1 |
| BRCA1 |  | transcription regulator |  | -1.847 | 1.75E-06 | IFIT2, IFIT3, IRF7, Ly6a (includes others), MX1, PLTP, S TAT1, TAP1 |
| STAT6 |  | transcription regulator | Activated | 2.041 | 2.90E-04 | FCGR1A, FCGR3A, IFIT3, Ly6a (includes others), Ms4a4b (includes others), Tgtp1, Trim30a/Trim30d |
| SATB1 |  | transcription regulator |  | 0.922 | 4.02E-03 | EPSTI1, IRF7, UBE2L6, XAF1 |
| SMARCB1 |  | transcription regulator |  | -0.429 | 4.63E-03 | FCGR1A, IGFBP2, MX1, OAS1,OAS3 |
| RARA |  | ligand-dependent nuclear receptor |  | -1.184 | 5.06E-03 | FOLR2, IFIT1B, OAS1, STAT1 |
| CEBPA |  | transcription regulator |  | -0.441 | 7.68E-03 | C3, FCGR1A, HMGA1, OAS2, PCSK6, S100G, TLR9 |

**Table S18**  **Summary of transcript cluster IDs common to ageing and hormonally manipulated mice.**

|  | **8-10 vs 28-30 weeks (n=75)** | | **8-10 vs 38-40 weeks (n=57)** | | **Late vs Early Ovariectomy 38-40 weeks (n=193)** | | **Vehicle vs P4 38-40 weeks (n=109)** | |
| --- | --- | --- | --- | --- | --- | --- | --- | --- |
|  | No. of transcript cluster IDs | P-value | No. of transcript cluster IDs | P-value | No. of transcript cluster IDs | P-value | No. of transcript cluster IDs | P-value |
| 26/8 weeks |  |  | 26 | 2.3x10^-56^ | 29 | 9.2x10^-47^ | 37 | 3.2x10^-75^ |
| 36/8 weeks | 26 | 2.3x10^-56^ |  |  | 31 | 5.1x10^-56^ | 33 | 3.7x10^-70^ |
| Cycling/  non-cycling | 29 | 9.2x10^-47^ | 31 | 5.1x10^-56^ |  |  | 41 | 1.8x10^-65^ |
| Control/p4 | 37 | 3.2x10^-75^ | 33 | 3.7x10^-70^ | 41 | 1.8x10^-65^ |  |  |

The P-values were calculated using the dhyper function in R (version 2.14.1).

**Table S19 Fold Change of Interferon Regulatory Factor 7 (IRF7) and the activation state predicted by IPA.**

| Comparison | Gene symbol of the upstream regulator | Fold change | Predicted activation state | Activation z-score | P-value of overlap | Number of transcripts |
| --- | --- | --- | --- | --- | --- | --- |
| 28-30 vs 10-12 weeks | IRF7 | -2.33 | Inhibited | -2.324 | 6.4E-16 | 10 |
| 38-40 vs 10-12 weeks | IRF7 | -2.08 |  | -1.53 | 2.69E-14 | 11 |
| Late vs early Ovx | IRF7 | -2.20 | Inhibited | -2.54 | 4.12E-11 | 13 |
| Vehicle vs Progesterone | IRF7 | -2.51 | * | * | 1.59E-28 | 22 |

The fold change was calculated using Cyber T and Rank Products for each of the comparisons listed. The predicted activation state was based on the direction of fold change of the associated transcripts downstream of IRF7. The P-value of the overlap was calculated by Fisher’s exact test with multiple testing correction. *z-score not provided by IPA.

**References**

Belteki G, Kempster SL, Forhead AJ, Giussani DA, Fowden AL, Curley A, Charnock-Jones DS & Smith GCS (2010) Paraoxonase-3, a putative circulating antioxidant, is systemically up-regulated in late gestation in the fetal rat, sheep, and human. *J Clin Endocrinol Metab* 95, 3798–3805.

Benjamini Y & Hochberg Y (1995) Controlling the False Discovery Rate: A Practical and Powerful Approach to Multiple Testing. *Journal of the Royal Statistical Society. Series B* 57, 289–300.

Blatti C & Sinha S (2014) Motif enrichment tool. *Nucleic Acids Res* 42, W20–5.

Carvalho BS & Irizarry RA (2010) A framework for oligonucleotide microarray preprocessing. *Bioinformatics* 26, 2363–2367.

Cheng C-W, Bielby H, Licence D, Smith SK, Print CG & Charnock-Jones DS (2007) Quantitative cellular and molecular analysis of the effect of progesterone withdrawal in a murine model of decidualization. *Biol Reprod* 76, 871–883.

Clark MB, Amaral PP, Schlesinger FJ, Dinger ME, Taft RJ, Rinn JL, Ponting CP, Stadler PF, Morris KV, Morillon A, Rozowsky JS, Gerstein MB, Wahlestedt C, Hayashizaki Y, Carninci P, Gingeras TR & Mattick JS (2011) The reality of pervasive transcription. *PLoS Biol.* 9, e1000625–discussion e1001102.

Cordeaux Y, Tattersall M, Charnock-Jones DS & Smith GCS (2010) Effects of medroxyprogesterone acetate on gene expression in myometrial explants from pregnant women. *J Clin Endocrinol Metab* 95, E437–47.

Jolma A, Yan J, Whitington T, Toivonen J, Nitta KR, Rastas P, Morgunova E, Enge M, Taipale M, Wei G, Palin K, Vaquerizas JM, Vincentelli R, Luscombe NM, Hughes TR, Lemaire P, Ukkonen E, Kivioja T & Taipale J (2013) DNA-binding specificities of human transcription factors. *Cell* 152, 327–339.

Mettus RV & Rane SG (2003) Characterization of the abnormal pancreatic development, reduced growth and infertility in Cdk4 mutant mice. *Oncogene* 22, 8413–8421.

Milligan SR & Cohen PE (1994) Silastic implants for delivering physiological concentrations of progesterone to mice. *Reprod. Fertil. Dev.* 6, 235–239.

Schroeder A, Mueller O, Stocker S, Salowsky R, Leiber M, Gassmann M, Lightfoot S, Menzel W, Granzow M & Ragg T (2006) The RIN: an RNA integrity number for assigning integrity values to RNA measurements. *BMC Mol Biol* 7, 3.

Telfer E, Gosden RG & Faddy MJ (1991) Impact of exogenous progesterone on ovarian follicular dynamics and function in mice. *J Reprod Fertil* 93, 263–269.
